# A Study and Analysis of Disease Identification using Genomic Sequence Processing Models: An Empirical Review

**DOI:** 10.2174/0113892029269523231101051455

**Published:** 2023-12-12

**Authors:** Sony K. Ahuja, Deepti D. Shrimankar, Aditi R. Durge

**Affiliations:** 1 Visvesvaraya National Institute of Technology, Computer Science and Engineering, India

**Keywords:** Genome processing, machine learning, disease, deep learning, gene network, classification

## Abstract

Human gene sequences are considered a primary source of comprehensive information about different body conditions. A wide variety of diseases including cancer, heart issues, brain issues, genetic issues, *etc*. can be pre-empted *via* efficient analysis of genomic sequences. Researchers have proposed different configurations of machine learning models for processing genomic sequences, and each of these models varies in terms of their performance & applicability characteristics. Models that use bioinspired optimizations are generally slower, but have superior incremental-performance, while models that use one-shot learning achieve higher instantaneous accuracy but cannot be scaled for larger disease-sets. Due to such variations, it is difficult for genomic system designers to identify optimum models for their application-specific & performance-specific use cases. To overcome this issue, a detailed survey of different genomic processing models in terms of their functional nuances, application-specific advantages, deployment-specific limitations, and contextual future scopes is discussed in this text. Based on this discussion, researchers will be able to identify optimal models for their functional use cases. This text also compares the reviewed models in terms of their quantitative parameter sets, which include, the accuracy of classification, delay needed to classify large-length sequences, precision levels, scalability levels, and deployment cost, which will assist readers in selecting deployment-specific models for their contextual clinical scenarios. This text also evaluates a novel Genome Processing Efficiency Rank (GPER) for each of these models, which will allow readers to identify models with higher performance and low overheads under real-time scenarios.

## 1 INTRODUCTION

Variables in both genetics and the environment have a role in the development of a number of common human diseases. These conditions include depression, schizophrenia, diabetes types 1 and 2, prostate cancer, and diabetes type 2. Discovering the genetic patterns that are connected with complicated diseases is of vital importance to the individuals who research and manage public health. It will assist us in gaining a deeper understanding of diseases and ailments that involve the interaction of several genes. The research into illness correlations has given conclusive evidence that several gene abnormalities are the underlying cause of complex disorders [1, 2]. Nevertheless, until very recently, isolating the particular genetic variations that are accountable for complicated diseases was a difficult operation that needed to be accomplished. Since the conclusion of the Human Genome Project [3, 4] and the beginning of the International HapMap Project [5], there has been a rise in the number of people interested in Genome-wide association studies (GWASs). The primary objective of this research is to identify Single-nucleotide polymorphisms (SNPs) that are associated with difficult diseases or characteristics such as diabetes. measurements (like a person's height) As of December 2014, it was shown that more than 15,000 SNPs have a genome-wide substantial connection with at least one illness or trait [6]. On the other hand, the majority of these studies merely give a sketchy outline of the genetic elements at play in complex disorders. Although research has discovered 18 SNPs that may raise a person's risk of getting Type 2 diabetes (T2D), these variations only account for around 6% of an individual's inherent propensity to the illness [7]. There are still many mysteries surrounding the heritability of diseases and traits, even in this day and age of scientific advancement. There is a discrepancy between the expected heritability of many common diseases, as determined by family and twin studies, and the total additive heritability, which is produced by adding up the effects of all of the SNPs that have been shown to be significantly connected with these disorders [7, 8]. When the effects of all SNPs that have been demonstrated to be substantially associated with these illnesses are combined together, it is shown that the overall additive heritability is considerably correlated with these diseases. Recent research [9] has shown that the bulk of heritability, which is not zero, may be accounted for by the combined effects of numerous genetic variations, each of which likely has only a little influence. This can be observed in Fig. (**1**) and was proved to be the case by combining the impacts of several genetic variants.

This is due to the fact that it is conceivable that each variation adds a small amount to the overall influence. The traditional test, which is based on a single locus, is unable to detect these changes. Furthermore, the number of groups of multiple variants to be analyzed in GWAS is large, which makes it hard to determine the influence that these variations have. This is owing to the fact that the gold standard test is based on a single gene as its primary component. Furthermore, due to the intrinsic randomness of GWAS settings, even little changes may have enormous sample connections. This is the case even when GWAS is used to analyze data. This is a shortcoming of the technique, and it has the potential to cause scientists to make incorrect conclusions about their findings from GWAS. However, because of the time and resources required for sample recruitment, a large sample size is often not feasible for a single GWAS.

Similar genetic risk variants have been observed in a broad range of difficult disorders [10-13] showing that there is a genetic relationship between these ailments. This shows that, in order to reveal the latent heritability of complicated disorders, it may be possible to eliminate erroneous connections, identify risk genetic mutations with modest implications, and undertake an integrated evaluation of linked genomic data. Innovative computer techniques for processing enormous volumes of data are of the utmost relevance in the field of biomedical research since high-throughput data collecting is rapidly becoming the industry standard. When doing an analysis of genetic information drawn from a large body of related research, it is helpful to have access to data on an individual level for all of the studies that were included. However, since there are restrictions on the sharing of data at the individual level, this may be difficult to do. In actuality, it is more typical to provide summary statistics, which often come in the form of P-values or z-scores. Combining P-values with Fisher's technique has become the most common approach to statistical analysis [14]. Researchers are hoping that by using this strategy, they would be able to discover significant SNPs that were present in all of the research that they looked at. Fisher's method was extended [15] so that weights could be taken into consideration. Additionally, P-values were included in the method. The inverse normal transformation was introduced [16], while Mosteller [17] improved upon Stouffer's method by taking into account weight when merging z-scores. Methods of statistical analysis that have been around for some time suffer from two significant drawbacks. To begin, it is important to note that despite the presence of numerous high P-values, a single P-value with a low probability might easily dominate the test statistic. In situations with a high number of dimensions and a limited sample size, statistical errors are more likely to occur owing to the fact that a number of seemingly little deviations will have a significant impact due to the influence of chance. Second, when P-values are averaged, any information about the family links between SNPs that may have been there in the initial data is lost. This is because averaging the P-values makes it impossible to determine which SNPs are related to one another. As a result of the fact that the majority of complicated illnesses are linked to several genetic variants, having this information is essential for comprehending the genetic architecture of these ailments. Biclustering analyses of summary statistics matrices are one method that may be used to uncover similar genetic patterns across several investigations. In these matrices, the columns represent the studies, while the rows show the genetic variants. An analysis of a matrix is required for this operation. The matrix has a list of studies along its columns, and a list of genetic variations along its rows. The bi-clustering method has undergone several iterations of refinement; for explanations of these refinements that are more in-depth [18-20]. Conventional bi-clustering methods do not perform particularly well on genomic data because of the enormous dimensionality of genomic data and the fact that the majority of the genetic variants contained in it are of little consequence. By applying a l1 penalty on the means of the bi-clusters, the recently developed statistical approach known as SparseBC [21] may produce bi-clusters that are both sparse and interpretable. The use of SparseBC in the processing of genomic data has many drawbacks, one of which is that it does not enable the creation of overlapping biclusters. This shortcoming may be directly attributed to the possible complexity of common genetic patterns found *via* GWAS. In addition to the overall genetic structure, the genomic data for each sickness or characteristic includes its own specific genetic variants. These variations are not shared with any other illness or feature.

A survey of such models along with their different internal characteristics is discussed in the next section of this text. Based on this detailed survey, readers will be able to identify optimal models for their application-specific use cases. Section 3 compares the reviewed models in terms of their quantitative measures, and also proposes evaluation of a new ranking metric, that can be used to identify models with overall higher performance under clinical scenarios. Finally, this text concludes with some contextual observations about the reviewed models, and also recommends methods to further improve this performance under real-time use cases.

## 2 DETAILED REVIEW OF EXISTING GENOME PROCESSING MODELS

A large number of genomic processing models for the identification of human body diseases are proposed by Researchers, and each of them defines an efficient & unique approach for the identification of different disease types. In this section, a detailed review of these machine learning based models is discussed, along with their approximate performance levels. Disease conditions are said to cause shifts in gene dependence networks [1]. The study of genomics focuses mostly on how networks change in disease states. Differential network analysis (DNA) is a popular topic for computer algorithm development, although most of these methods have certain data requirements. High throughput technologies allow for several measurements of gene activity to be taken at once. There may be commonalities and distinctions across these data formats. It is important to find new ways to compare networks while working with data in different forms. In this research, researchers combine information on gene expression and mutations to identify cases of gene network rewiring. When comparing two sets of data, a group bridge penalty function is used to determine what is the same and what is different. Their strategy has proven superior to competing approaches in simulated environments. Ovarian cancer patients who develop resistance to platinum may have their mutations in the underlying gene networks detected using their technique. The data types that share an edge with each other are not always the same. Their technique allows us to infer hub genes in many networks that control therapeutic resistance in ovarian cancer.

The identification of shared molecular pathways across illnesses is crucial for improving disease prognoses and developing targeted treatments [2]. The common disease genes causing metabolic illnesses are elusive due to the complexity of metabolic pathways. Complex bioinformatics models that make use of both biological data and computational tools are needed for this purpose. To find the genes in common between metabolic syndrome (MS), (T2D), and coronary artery disease (CAD), researchers used a network analysis technique. Also, Artificial intelligence methods work best for cardiovascular genetics [22]. Using a large amount of publicly available information on gene expression, protein-protein interactions, and gene ontology, Researchers constructed weighted gene co-expression networks. Using MCL, SPICi, and Link-comm graph clustering (GC) methods, Researchers were able to determine the relevant modules for 90 distinct disease network topologies. Researchers also evaluated illness modules to identify the most biologically sound approach. By superimposing disease modules, researchers were able to identify 22 genes that were shared by MS-CAD and T2D-CAD. In addition, earlier scientific studies found that 19 of these genes were connected to major illnesses in some way. This work demonstrates the utility of combining data from diverse biological sources with computational methods for locating disease-causing mutations, and it sheds light on the genetic pathways shared by a wide range of metabolic disorders.

Experts in genomics endorse research [3] that stresses the need to identify disease-causing genes in order to fully understand an illness and work toward a treatment. Several computational methods for identifying disease-causing genes have emerged in recent years. It is still difficult to improve disease gene prediction accuracy using disease and gene-related data like gene ontology and protein-protein interactions. Here, Researchers set up the HerGePred framework for utilizing Heterogeneous disease-related gene (HDGN) embedding representation in disease gene prediction (called HerGePred). In this setting, researchers can build an LVR of HDGN nodes, which is a representation with a reduced number of dimensions. Researchers then propose two algorithms, an LVR-based similarity prediction and a random walk with restart on a reconstructed heterogeneous illness-gene network, to reliably identify disease genes (RWRDGN). Researchers begin by exploring the overlap distribution of illnesses based on their degrees of similarity, and then Researchers conduct an experiment to prove the framework's assumptions. According to the results of the study, the LVR of nodes successfully maintains the HDGN's local and global network topology. Researchers then compare the performance of their methods to that of established disease gene prediction algorithms by employing tenfold cross-validation and external validation. The experimental results show that the RW-RDGN performs better than the state-of-the-art techniques. In order to analyze molecular pathways and perform experimental validation, it is essential to have results from the prediction of disease candidate genes.

To perform medical research, understanding the molecular basis of diseases, and analyzing disease function, it is essential to construct a network of disease-disease similarities [4]. Using a robust protein interaction network and numerous pathway datasets, researchers build a Human Pathway-based Disease Network (HPDN) to investigate the connection between diseases and their underlying connections. Their analysis reveals a strong correlation between the number of overlapping gene sets, the degree of gene set overlap between two disorders, and the number of common functional pathways between them. Both HPDN and illness networks based on genes and symptoms coexist in the real world. HPDN is able to foretell fresh disease-disease connections by using a large scientific literature library and the CTD benchmark. Systems medicine has the potential to solve major problems, like drug redistribution, by focusing on routes. Their network's dense edge structure suggests that Prednisone and folic acid may have separate indications for serious illnesses.

Disease gene prediction is a challenging issue with applications in early diagnosis and the creation of therapies, as stated by genomic experts [5]. The vast majority of disease gene prediction algorithms do not use patient-specific clinical data. Here, researchers provide a strategy for predicting disease genes (dgSeq) by combining information from Protein Protein Interaction (PPI) networks, clinical Ribonucleic Acid (RNA-Seq) Data, and Online Mendelian Inheritance in Man (OMIM) data. Using RNA-Seq rewiring data, their dgSeq creates differential networks. In order to create a disease-gene network using OMIM data, researchers must first filter out all of the healthy genes (negative samples). Logistic regression (LR) classifiers are then trained once differential and PPI network analysis is completed. Their dgSeq has an AUC of 0.88 for detecting breast cancer genes, 0.83 for detecting thyroid cancer genes, and 0.80 for detecting Alzheimer's disease (AD) genes. Gene set enrichment and anticipated results suggest that dgSeq may be useful for finding new disease genes. Current machine learning methods rely on skewed data due to the imbalance between the number of known disease genes (positive samples) and unknown genes (negative samples) [23].

Measurement of disease correlations is crucial in modern biology and medicine, according [6] to the study. The discovery of disease connections has the potential to enhance disease diagnosis, medication repositioning, and drug development. Many methods for comparing illnesses have been developed during the last decade thanks to the availability of high-throughput biological data. Protein Protein interaction networks (PPIN) are seldom included in these methods. It has recently been shown that the PPIN region is a hub for interaction between genes and proteins that are linked to a certain illness. Here, researchers present ModuleSim, an innovative approach to assessing illness associations using information from gene-disease association studies and the idea of disease modules. Disease Ontology(DO) classification is strongly correlated with ModuleSim's disease similarity, disease module linkages, and disease module modularity. ModuleSim outperforms four popular methods by analyzing disease-gene and PPIN data to reveal causal links between diseases. ModuleSim's capacity to discover illness connections is highlighted by its disease similarity network.

Researchers [7] who study the human genome assert that familiarity with the connections between phenotype and genetics is essential for comprehending the origins of illness. There has been a lot of work invested into creating reliable computational methods for predicting disease genes. Due to the quantity and complexity of medical data, it is challenging to build a deep neural network model to discover disease genes. Researchers built a model that uses a deep neural network to combine symptoms and genotypes in order to identify disease-causing genes (termed PDGNet). Their approach employed training samples and information from several perspectives on disease and genes to fine-tune the parameters of a deep neural network and produce a deep vector of disease and gene properties. Compared to the state-of-the-art method, PDGNet fared better during the course of extensive testing sets. A large number of links were found between candidate genes, and their functions were shown to be quite similar. Researchers used externally curated data and published medical literature to verify the top predicted genes for Parkinson's disease, showing that the candidate genes had a high probability of influencing the selection of causal genes in the “wet experiment.”

Genomics Researchers report that ncRNAs are increasingly being used as diagnostic tools [8]. Predictions of disease-associated ncRNAs are useful for biological screening. It is challenging to integrate data characteristics due to the wide variety of ncRNAs present in the human genome. Existing methods may have trouble getting going from a cold start. Using experimentally verified disease-gene correlations, this work introduces a resource-allocation-based approach (RAA) for predicting disease-associated ncRNAs. Possible ncRNAs were ranked based on their relevance, as determined by the values of available resources. Previous leave-one-out cross-validation methods for 537 diseases were improved by their method. By showing that the majority of the best-predicted outcomes were validated by external sources, case studies on three serious diseases demonstrated the efficacy of their method. The release of extensive data enables the biological identification of ncRNAs with a link to illness. Noncoding RNAs (ncRNAs), including microRNA (miRNA), long ncRNA (lncRNA), and circular RNA (circRNA) are important regulators, regulating the function of mRNAs directly or indirectly [24].

Experts in genome processing have noted a recent uptick in cases of diabetes mellitus. Ninety percent of people with diabetes have type 2 diabetes [9]. Disease results from a complex interplay between genetic predisposition and environmental influences. Determining the underlying genetic cause of a disease is critical for both diagnosis and treatment. Complex diseases are examined in depth using computer-based network techniques, such as the identification of likely disease genes. Researchers present a bioinformatics framework for ranking genes on bilayer biomolecular networks associated with T2D using a tweaked version of the PageRank algorithm. Differential mutual information (PRDM) is used to weight the networks in order to assess the contextual specificities between genes and proteins in transcriptomic and proteomic datasets. In order to establish a hierarchy in the bilayer network, Researchers prioritized the genes involved in diabetes relative to all other genes using an improved PageRank method. Researchers show that genes associated with established diseases consistently outperform those associated with healthy populations. These highly ranked genes have been linked to type 2 diabetes risk and malfunction *via* functional studies.

One new instance of Alzheimer's is identified every 3.2 seconds, meaning that there are more than 10 million new cases of Alzheimer's each year [10]. AD is caused by both genetic and environmental factors; that affect the brain of a person over time. The combination of machine learning and image processing might lead to the diagnosis of (AD) [25]. Here, researchers have used ML methods to classify AD using gene expression data and images. In the beginning, MRI pictures were put into categories using SpinalNet and CNN. Researchers categorized diseases with microarray gene expression data and the classifiers KNN, SVC, and Xboost. Previous methods relied either primarily on visuals or gene expression, but Researchers combined the two and provided an explanation. It was not apparent how disease and gene classification systems worked; the results of these classifiers should be well-supported. Researchers employed explainable AI (XAI) to build robust forecasting models. Researchers employed locally interpretable, model independent (LIME) explanations to build XAI. LIME also examines how genes were predicted and are responsible for AD patients. In the case of image data, the accuracy of CNN is 9.76% higher than that of SpinalNet. When evaluating gene expression data, SVC increases accuracy. The LIME diagram in Fig. (**2**) shows how genes were discovered for an individual with AD by analyzing gene expression data samples.

Increasing evidence suggests that long noncoding RNAs (lncRNAs) have a significant role in biological processes and the emergence of human diseases, as shown by a genetic analysis published [11]. Understanding how to foresee correlations between lncRNAs and complex human illnesses is of paramount importance. These methods often focused on only two networks (lncRNA and disease) while ignoring the others. In this investigation, researchers built a multi-layer network by connecting previously established lncRNA-disease, lncRNAs-gene, and disease-gene association networks with lncRNA, disease, and gene similarity networks. Then, researchers built the MHRWR model to foresee possible lncRNA-disease connections using a random walk with restart. The efficiency of MHRWR was evaluated based on experimentally shown lncRNA-disease associations. Previous methods could not compete with MHRWR's 0.91340 AUC. Researchers used MHRWR to validate related lncRNAs in case studies of colon, colorectal, and lung adenocarcinomas to show that their results were reproducible.

Several risk loci for (AD) have been discovered by GWAS, but heredity and interpretability hurdles must be cleared before the causal genes in these risk loci can be identified and the mechanism of AD can be explained. This study [12] aimed to identify the underlying genetic causes (AD) by first identifying risk loci and then causal genes. Researchers combined the results of several different prediction methods (DAPPLE, DEPICT, Prix Fixe, *etc*.) that make use of GWAS data, such as gene functions, protein-protein interaction networks, co-function networks, or expression QTL data. There are now 43 potential AD genes, 8 of which have a high degree of certainty (BIN1, CR1, CLU, HMHA1, MS4A4A, MS4A6A, PICALM and PVR). The final tally of mapped causal genes stands at 43. The importance of lipid/lipoprotein homeostasis in AD was recently highlighted by a Gene Ontology analysis that identified the underlying genes responsible for the illness as being heavily involved in complexes and processes linked to lipids and lipoproteins. These causal genes were shown to have distinct spatiotemporal expression patterns, indicating that they fulfilled a variety of functions across various cell types and developmental stages. Patients with AD showed dysregulation of the top eight causal genes, indicating their involvement in the genesis of the illness compared to controls. Their research results may provide some insight into the etiology of AD. Finding urgent treatment targets will help validate the role of the genes suspected of causing AD.

Genomic researchers [13] have shown that the causes of vitiligo are intricate. Understanding the cause of vitiligo is challenging. In the first step, researchers used gene ontology and protein interaction network keywords to identify proteins that could have a role in illness. It has been shown that 109 proteins have a role in either the onset or the development of disease. Proteins associated with vitiligo illness were afterward prioritized by comparing their characteristics to conventional target identification criteria. A novel method for evaluating the targetability of disease-associated proteins was offered, which combines historical data with efficient techniques. 68 medication-related vitiligo goals were defined using digital resources and scripting. This list recommends potential new targets and is in line with the research community's current focus on certain proteins; however, it may be tailored to a user's individual situation by changing the weights for the selected criterion (*i.e*., a quantitative approach) or the criteria under consideration (*i.e*., a qualitative approach).

Scientists who specialize in analyzing genomes state [14] that understanding a disease's genetic basis is essential. Genes and genetic diseases are a major cause for worry when it comes to human health. Experiments on several candidate genes are required for the finding and linking of disease genes, but they are both time-consuming and expensive. In order to find a disease's candidate gene quickly and cheaply, scientists might turn to computational approaches. Because the sequence or network properties of protein-protein interactions of genes responsible for the same or related disorders are less varied, the majority of these tactics rely on phenotypic commonalities. These methods, which are exclusive to a particular gene-disease relationship, find links between genes and diseases by using basic network properties, topological aspects, gene sequence data, or biological parameters. In their research, Researchers develop and analyze cutting-edge computational methods for identifying genes that are causal to illness. Researchers isolate putative genes while ignoring certain biological and topological constraints. DisGeNET proves a knowledge platform for disease genomics [26]. Researchers use DisGeNET's disease-gene association data to compare and contrast different computational methods using 10-fold cross validation. After being combined with improved feature sets, certain computational methods achieve higher accuracy (up to 93.8%), recall (up to 93.1%), and F-measure (up to 92.2%) than state-of-the-art approaches. Researchers apply their methodologies to the study of thalassemia, diabetes, malaria, and asthma. The results of DELM's simulations show that it is more accurate than previous methods.

Experts in genomics have speculated [15] that T2D may increase the likelihood and severity of other illnesses, including musculoskeletal issues. Comorbidity interactions cannot be thoroughly explored using traditional endocrinological techniques. Researchers identified common disease pathways by analyzing tissue transcripts. Low peak bone density is an important predictor of osteoporosis, therefore researchers investigated RNAseq and microarray transcript datasets from T2D and chronic bone and joint illnesses such as JIA, OA, and RA. Subjects with and without the disease are included in these data sets. All condition’s Database of Essential Genes (DEG) are compared to T2D DEGs. Data from pathway studies and gene ontology techniques were used to look for associations between overlapping DEGs and diseases in the SNP-disease linkage (dbGaP) and gene-disease association (OMIM) databases (those shared by T2D and a bone or joint problem). To learn more about DEG-Transcription factors (TF) and DEG-microRNA (mi-RNA) interactions, researchers analyzed gene targets of TF and miRNAs. SYK, UCP3, ROR1, PPARG, BUB1, AKT2, ADCY2, and CCR5 were all plausible candidates in commonly used pathways. DEG-TF and DEG-miRNA interactions networks identified TFs (GATA2, FOXC1, USF1, YY1, E2F1, JUN, RELA, CREB1, TFAP2A, NFB1) and miRNAs (mir-335-5 p, mir-16-5 p, mir-124-3 p, mir-218-5 p, mir-98-5 p, mir-29b-3 p, mir-3135 b, mir-29). Using a data-driven approach, researchers were able to pinpoint innovative methods for counteracting pathogenic processes by identifying and confirming regulatory components and cellular pathways *via* which T2D may affect bone and joint disorders.

Geneticists agree that determining the relationships between illnesses is essential for understanding their genesis, pathophysiology, and clinical manifestations [16]. Many methods for comparing diseases rely on gene interactions or ontological disease notions. These methods are heavily reliant on the availability of certain datasets and ontologies. Diseases from a single data source are compared using a single parameter in several methods, which might result in misleading inferences. To achieve this goal, researchers presented an ontology-free method, namely RADAR, for discovering shared disease representations using machine learning. A network for disease similarity was built by establishing links between biological entities associated with different diseases. The creation of a multi-layer disease similarity network was accomplished by combining existing disease similarity networks from different data sources, and representation learning was utilized to conduct an analysis of disease similarities. One hundred randomly selected disease sets were used with a reference sickness set to assess RADAR's efficacy. Clinical trials suggest that RADAR can detect illnesses with similar symptoms effectively.

Understanding cell differentiation, drug discovery, and disease etiology all rely on the identification of important genes in comparative states (EGS). In this article [17], researchers suggested a method called Prediction of Essential Genes in Comparison states (PreEGS). PreEGS compiles topological and expression features of each gene to facilitate comparisons across network states. PreEGS uses a strategy of expanding positive samples to level out the distribution of positive and negative samples. For best results on synthetic data, researchers settled on a PreEGS variant based on the random forests model (PreEGSRF). Three additional methods that relied on machine learning were compared to PreEGSRF and the other six methods aim to predict EGS. A gene regulatory network (GRN) describes the hierarchical relationship between transcription factors, associated proteins, and their target genes [27]. PreEGSRF analyzed four gene regulatory networks and identified five key Leukemia genes along with five enriched KEGG pathways. Leukemia is associated with the four major genes found and with all the predicted pathways. Identifying disease-causing genes, driver genes for cell fate decisions, and other indications of complex biological systems is possible using PreEGSRF.

Clinical decision support, medication, comparing data across time, and evaluating diagnoses are only some of the applications for disease ontologies that have been curated by humans [18]. These ontologies assume commonalities in the anatomy or histology of various diseases in order to classify them. Despite advances in molecular biology, disease ontologies have maintained a “reductionist” approach. Disease diagnosis, the identification of illnesses with similar pathobiology levels, and the creation of new medicines all benefit from the proximity connections of disease modules (DMs) in the human interactome network. Inadequate disease-gene correlations and the inability to depend on clinical studies to test the validity of the similarity linkages produced by the structural similarity of DMs are only two of the drawbacks. In order to shed light on disease similarities, this study investigates the correlation between the closeness of illnesses in human-curated ontologies and the structural proximity of related DMs in the interactome. In this paper, Researchers provide a method (and associated algorithms) for automatically constructing a hierarchical structure from DM proximity contacts and evaluate it in light of a disease taxonomy chosen by humans. Researchers provide examples of how the proposed method could be used to enhance and broaden existing classifications of human illnesses, identify promising network regions for the identification of new disease-gene relationships, and investigate the structural and phenotypic similarities between various medical conditions.

Molecular networks [19] encompass a wide variety of biological and functional linkages between genes and gene products, which may contribute to the identification of novel genes and pathways for a certain disease phenotype. Although there have been significant advancements in high throughput interactome mapping, dense Gene Networks remain elusive due to insufficient gene connections (GNs). By bringing together six genome networks: STRING, ConsensusPathDB, HumanNet, GeneMANIA, GIANT, and BioGRID, researchers proposed a framework called Neural integration of heterogeneous data (NIHO) which improves and rounds out GNs. Using neural networks and matrix completion, NIHO compiles gene features from disparate data sources. Next, the acquired low-dimensional representations are used to estimate the spatial closeness between genes. For the purpose of inferring gene associations, NIHO calculates proximity scores and appends them to GNs. Improvements in GNs' capacity to retrieve disease gene sets after NIHO processing. To add, researchers discovered that NIHO functions best in highly diverse networks.

Genomic Researchers claim that prioritizing disease genes is essential for network medicine [20]. In theory, interactome modules categorize disease-related genes. In this paper, researchers offer DIAMOnD Background Local Expansion (DiaBLE), an improved variant of the connectivity-based method DIAMOnD. DiaBLE's gene universe is the minimal local extension of the current seeds at each iteration step. DiaBLE enhances the DIAMOnD gene prioritization scores by increasing their biological consistency and cross-validation. However, comparing different methods of gene prioritization is not the focus of this study. Finally, researchers examine DiaBLE's effect on two distinct cancers (head and neck squamous cell carcinoma and kidney renal clear cell carcinoma).

According to genomics specialists, the solution to the drug development and discovery bottleneck is drug repositioning or finding new uses for existing medications [21]. *In silico* methods have been developed to anticipate medication-disease connections, which may help in drug repositioning. In theory, a meta-paths-based strategy may reach the same level of performance as more traditional methods while using far less data. As it stands, current meta-path-based approaches just count retrieved routes and leave out intermediate node information like proteins linked to diseases and treatments. Here, Researchers provide an ensemble learning strategy for predicting drug-disease association (DDA) using Meta-path Gene ontology Profiles (MGP). Gene ontology (GO) terminology is used to draw connections between medications and illnesses and the activities they affect in a drug-GO-disease network. Each drug-disease combination in MGP-DDA comes with a corresponding GO profile based on meta-paths. Classifiers based on bagging and boosting are developed to separate positive (already established) drug-disease connections from negative (yet-to-be-discovered) ones. With an accuracy of 88.6 percentage points higher, MGP-DDA dominates state-of-the-art methods. Their drug repositioning method has broad applicability, as shown by the MGP-finding DDA's of 37.7% more unique medication-disease linkages when utilizing ClinicalTrials.gov data.

Deep learning algorithms say genomics experts [28], need a large number of training samples to successfully reveal hidden patterns in data and give better outputs. Fewer patient samples are represented in the omics data generated by high-throughput sequencing for brain illnesses (tens to hundreds of samples). The small sample size precludes using convergent gene sets in statistical or machine learning biomarker selection methods. Mathematical approaches for detecting biomarkers have variable levels of success depending on the data collection. To get around this problem, Generative adversarial network (GAN) architecture might be used. The discriminator's accuracy and stability may be enhanced if the generator supplies samples with distributions that are consistent with those in the training set. A unique adversarial network model was developed in this study by combining a denoising auto-encoder (DAE) generator with a multilayer perceptron (MLP) discriminator. The probability distribution was modified as a result of the residual prediction error being backpropagated to the DAE decoder. Researchers used this method to build a platform for using RNA-seq data to predict disease-causing genes. When compared to conventional methods, deep learning is much more effective in locating disease genes. Insight into the genetic basis of illness symptoms was gained by an analysis of experimental data that uncovered novel disease-related genes and brain circuits.

According to [29], temporal patterns of gene expression have garnered a lot of interest in the quest to understand cancer's genesis and development. Insufficient sample sizes and improper data processing were common in analyses of gene expression patterns. Because of these methods, tracing the origins of cancer is more difficult than ever. The gene expression profiles of 556 and 566 colorectal cancer tissues were analyzed in this research. The linear mixed-effects regression model (LMER) was used to identify differential gene expression according to cancer staging. The identification of two distinct patterns of gene expression that shift as cancer develops. The cell cycle and metabolism are regulated by certain genes, while the nervous system and development are influenced by others. Furthermore, Researchers analyzed gene networks. To better understand the development and progression of cancer, researchers recommend using gene expression profile analysis. By using this method, researchers can learn more about the development of cancer.

The biomedical community's attention remains fixed on the investigation of human disease-pathway interactions [30]. Possible insights into disease mechanisms or interactions may be gained by examining the connections between diseases and the channels *via* which they spread. Disease diagnosis remains imprecise after decades of research. A computational model of disease-pathway associations is presented in this research paper. The presented model incorporates PageRank (PR) and Random Walk with Restart on heterogeneous network (RWRH). The disease-pathway connection model developed by RWRH allows for the prediction of such relationships. Pathologists may benefit from using the model because it may help them make sense of the connections between diseases, treatments, and patient outcomes. Researchers used a pathway-based strategy to investigate genetic alterations and illness variance. A biological network was built to better understand illness etiology by using the shared gene connections of disease pathways. The process of building the network was split in half. First, researchers determined how similar the networks were. Secondly, researchers constructed and correlated a network of similarities between disease-disease (DD). Researchers looked into the role that high-PR nodes play in spreading disease and establishing new routes. Researchers looked for links between diseases and analyzed them. Researchers used a disease-pathway bipartite network based on pair-wise similarity of sequence expression weights to integrate biological data. Disease-pathway prediction scores were calculated using these weights and the multilayer resource allocation method. Researchers looked at a 210-by-1855 matrix, where the pathways were in the 1855 columns and the pathologies were in the 210 rows, using a leave-one-out cross-validation method. The matrix contained 13,838 disease-pathway links. The best predictors were a precision-recall curve for the two classes with an area under the curve (AUC) of 0.8218. These outcomes demonstrate the method's superior performance. Researchers used prior connections and literature searches to hypothesize links between pathogens, DDs, and disease pathways.

Genome Researchers [31] found that state-of-the-art selection approaches fail to detect subtle but cumulative effects in high-dimensional omics datasets. In certain disorders, these elements play a role. In a three-stage process, Netboost decreases dimensions. The network's critical edges are first identified using a topological overlap measure and boosting-based filter. First principal components are utilized to aggregate module data after the use of sparse hierarchical clustering (PCA SHC) to find modules. Using cross-validated prediction error curve estimates, researchers show that using Netboost in conjunction with CoxBoost improves prediction performance over variable selection and a different clustering method when analyzing DNA methylation and gene expression data from 180 patients with acute myeloid leukemia (AML). The signature of chromatin-modifying enzymes was also found in the unrelated dataset AMLSG 12-09. In a second use case, the classification of Huntington's disease-related RNA-sequencing data is boosted when Netboost is used with Random Forest classification. Use of the Bioconductor package in R as a dimensionality reduction and hypothesis generation tool, Netboost is particularly useful in omics-related applications.

A number of time-varying differential networks covering many cancer stages are recommended by the study [32]. Gene mutations may cause a restructuring of these networks, which might lead to the sharing of hub nodes. Different methods have been devised to estimate differential networks from gene expression data, but most of them fail to account for commonalities across such networks. Researchers provide an efficient & unique Gaussian-based graphical model (GGM) for estimating several time-varying differential networks for cancer network rewiring simultaneously. D-trace loss must be used when computing differential networks. Having a form similar to a tree the lasso penalty finds nodes that are hubs in several networks and those that are hubs in just one. Their solution consistently outperforms state-of-the-art approaches in simulated environments. Their study uses data from The Cancer Genome Atlas to investigate how breast cancer-related gene networks change over time. Using predicted differential networks, researchers find previously identified genes that have a role in breast cancer. For breast cancer disease artificial intelligent tools work best for its genetic data [33].

During and after transcription, small noncoding RNA molecules called miRNAs affect the stability of genes and the rate at which they are translated [34]. Disease in humans has been linked to dysregulation of miRNA targets. Correctly predicting where miRNAs will be used is a key step toward developing treatments for human diseases. There have been recent presentations of computational methods for predicting miRNA targets. The intricate relationship between miRNAs and their targets makes these methods vulnerable to false positives. High-precision data processing is now possible thanks to the growing number of experimentally confirmed miRNA targets. In this article, a novel recommendation system model for miRNA target prediction is built using a new matrix completion technique (miRTMC). The miRNA-gene interaction network, gene similarity network, and miRNA similarity network are all brought together in the miRTMC. The latent determinants of whether a gene is a miRNA target are assumed to be highly interconnected, leading to a low-rank adjacency matrix in the heterogeneous network. Their next step is to employ a linear least squares model with nonnegative constraints that make use of the nuclear norm regularization. The alternating direction method of multipliers (ADMM) provides a quantitative solution to the problem of incomplete matrices. Their findings show that MiRTMC outperforms competing methods across a range of metrics.

Genetic experts assert [35] that COVID-19 is a highly contagious illness caused by severe acute respiratory syndrome (SARS-CoV-2). Both the elderly and those with conditions like diabetes, cancer, or cardiovascular disease had a higher case-fatality rate. SARS-CoV-2 proteins communicate with their human counterparts, including ACE2, TMPRSS2, and BSG. Researchers created a virus detection capable protein-protein interaction sub-network using these three proteins as seed nodes. Diabetes, cancer, and cardiovascular disease were linked to insulin resistance, AGE-RAGE signaling in diabetic complications, and adipocytokine signaling. The underlying molecular mechanism of COVID-19's lethality is linked to aging and associated diseases. Gene expression investigations have led to the discovery of drugs that interfere with certain proteins/pathways. Researchers zeroed attention on drugs that significantly inhibit key proteins like ACE2. Entinostat and mocetinostat were tested for non-small-cell lung cancer, however, COL-3 was effective for treating acute lung injury and respiratory distress. Researchers suggest that recycling these medications can be helpful for COVID-19 therapy.

According to studies [36], circular RNA (circRNA) is a kind of non-coding RNA that is produced naturally inside the body. CircRNAs seem to have an important role in a wide variety of biological activities, as shown by the literature. More circRNAs can be uncovered using high-throughput sequencing methods, but doing so is time-consuming and costly. Here, researchers detail a computational strategy for predicting circRNA-disease connections from a variety of data sources by utilizing metapath2vec++ and matrix factorization (called PCD MVMF). Building reliable networks calls for attention to a number of different issues. Disease-related genes and semantics are used to generate semantic and functional similarity networks once circRNA annotation, sequencing, and similarity networks have been built. Metapath2vec++ is used on a combined heterogeneous network to learn embedded characteristics and an initial prediction score. Researchers apply similarity-constrained matrix factorization to get the final forecasts. The leave-one-out, five-fold, and f-measure tests are used to assess PCD MVMF. The higher prediction power of PCD MVMF is shown by these assessment indicators. Common health problems have been used to prove PCD MVMF's effectiveness in research. PCD MVMF might be used to predict circRNA-disease associations.

Scientists confirmed [37] that decades of study have shown that disease-associated genes are to blame for the malfunctioning of biological systems, which leads to complex disorders. Over the last two decades, there have been several efforts to examine various interaction networks in an effort to find disease-related gene modules. Numerous existing approaches ignore disease-specific sample features, making it difficult to locate a sizable disease-related module. For the goal of isolating disease subsystems, this research proposes an Evolutionary optimization technique (EOT) with many objectives. For each disease sample, the proposed technique applies a multi-objective genetic algorithm to optimize the module's correlation with the disease and its intra-link density. Gene expression data for asthma show that the suggested strategy is superior to other methods for identifying disease modules. The selected disease module is then used to classify illness and control samples, hence decreasing the rate of misclassification for real-time scenarios.

According to [38] studies, it is still challenging to determine a patient's clinical prognosis on genetic information alone, especially when it comes to diseases like AD and heart disease. Pattern recognition algorithms may be able to reliably forecast disease when GWAS finds strong associations between genetic predictors (such as SNPs) and health outcomes. It is possible to employ latent multivariate interactions of genetic predictors in signal processing. Predicting clinical outcomes using genetic patterns is the focus of this essay. Researchers show that genomic data may be improved upon using multiresolution transformations by identifying multivariate interactions and, in some cases, even surpassing the performance of standard clinical outcome prediction algorithms. While comparing with training logistic regression on raw SNP data, training logistic regression on multiresolution spaces (LRMS) may result in a 6-percentage point improvement in area under the ROC curve as it incorporates a multiresolution analysis block as a pre-processing step as shown in Fig. (**3**) for predicting late-onset Alzheimer’s disease(LOAD).

According to a study [39], predicting genetic aberrations is a difficult problem in the field of biology. Malignant diseases such as cancer, diabetes, cystic fibrosis, dementia, and leigh syndrome all have their roots in chromosomal anomalies. Many explanations and ideas have been proposed for why genetic abnormalities could occur. The scope of genetic data expanded to encompass the protein sequence, and advances in AI have allowed for the application of machine learning and deep learning to predict genomic anomalies. The advent of deep learning coincided with the advent of machine learning. In the past, researchers have used supervised, unsupervised, and semi-supervised learning methods to make predictions about genetic illnesses. The vast bulk of research that used genetic sequence data expected binary difficulties. The predictions made by these methodologies were questionable because of their low levels of accuracy and their reliance on binary class prediction techniques. Most approaches relied on RNA gene sequences, which made data organization challenging. This study proposes the creation of an Advance genome disorder prediction model (AGDPM) for predicting genomic multiclass disorder by using a vast quantity of data and AlexNet as an effective convolutional neural network architecture. When compared to AlexNet, AGDPM achieves higher accuracy in both training and testing (90.89% *vs*. 81.25%). The state-of-the-art genome disorder prediction model uses multiclass prediction to reliably foretell cases of genetic disease and evaluate voluminous patient datasets. Single-gene, mitochondrial, and multifactor gene inheritance problems may all be predicted using AGDPM. In biomedical studies, AGDPM will improve mortality rate management and genetic condition prediction.

Researchers [40] claim that molecular biology has uncovered the causes of human illness, however, this is not the case. To better prevent, diagnose, and treat disease, it is helpful to have a better understanding of its molecular basis. Over the last two decades, techniques like positional cloning and GWAS analysis have been used to learn more about the connections between genes and diseases. Diseases have been shown to be linked to coincidental clinical conditions. There is hope that the identification of disease-related genes that are commonly held among humans would facilitate the assessment of disease similarities, the identification of genetic links, and the development of a network of human diseases. Despite the availability of a number of approaches to illness similarity measurement, these approaches only consider genes or functions that are independently related to diseases, ignoring gene-function connections. A consequence of this is a change in how illnesses are categorized. Researchers utilized a network-based sickness module (NBS) to investigate possible gene-activity connections among human disorders. In this article, the 299 illnesses are sorted into 15 categories according to their separation score. Disease-related gene annotations, GO terms, and KEGG pathways were used to determine the most effective clustering method. Fig. (**4**) shows the entire analysis procedure. Differentiating between groups of diseases was made easier with the help of the found signals. This research also provides a novel approach to foreseeing network and function characteristics.

The potential impact that machine learning will have on medical diagnosis is discussed by genomics experts [41]. SNPs are a major contributor to the genetic diversity of humans and have been linked to a variety of illnesses. Using SNPs, researchers can separate diseased samples from healthy ones. Excellent classification accuracy in a high-dimensional space is essential for diagnosis and therapy. In this study, the authors provide a precise hybrid feature selection strategy for determining the best SNP subset. The suggested technique uses recursive feature removal from Conditional Mutual Information Maximization (CMIM) and Support Vector Machine (SVM). Comparisons of the proposed method's performance with that of four state-of-the-art feature selection strategies-minimum redundancy maximum relevancy, fast correlation-based feature selection, CMIM, and ReliefF-were conducted on five SNP data sets from the gene expression omnibus genomics data repository at the National Center for Biotechnology Information. The experimental results show that the selected feature selection process beats all other strategies tested, resulting in a 96% accuracy rate in classification on the provided data. These results demonstrate the potential use of whole-genome SNPs for discriminating between individuals with and without complicated illnesses.

Complex network theory [42] may be used to study the massive human protein interaction network (HPIN) after the human genome project is finished. Proteins are the products of genes. Genes that are alive and well, disease-resistant, low-maintenance, housekeeping (HK), and enriched in tissues (TE) are essential. Through networks, these genes are able to interact with one another. Two large-scale HPINs and six smaller subnetworks are compiled using databases and other sources of available information. In general, HPINs and their constituent subnetworks are modular, sparse, small-world, scale-free, and have assortative difference levels. In Hong Kong, the Hong Kong sickness subnetwork, and the significant illness subnetwork are the most linked groups of nodes. Statistical analysis of the HPIN's topological structures revealed visual differences across HK, TE, lethal, and conserved genes. Receiver operating characteristic (ROC) curves can distinguish between necessary and non-necessary genes 70% of the time. Closeness, semi-locality, and eigenvector centralities can distinguish HK from TE genes with 82 percent precision. Cancer genes, HK disease genes, and TE disease genes all have distinctive visual characteristics, as shown by disease gene classifications, cluster dendrograms, and Venn diagrams. The findings are useful for finding functional genes by using topological structures. Competitive interactome features were uncovered, which may have implications for networked medicine and the management of biological networks.

Disease susceptibility can be determined in the human genome, but only *via* the discovery of epistasis [43]. In order to identify epistasis, Multifactor Dimensionality Reduction (MDR) is a useful tool. The high-risk (H) and low-risk (L) categories have not been thoroughly investigated in MDR operations. In order to enhance binary classification, the authors of this study suggest using a fuzzy c-means-based entropy (FCME) approach. An FCMEMDR membership was used in this tactic. The discrimination of multifactor genotypes with possible epistasis was enhanced by linking FCME and MDR. Researchers used MDR measures of classification rate and Likelihood Ratio (LR). Two FCMEMDR measures were shown to have higher detection rates than prior MDR-based approaches across a number of synthetic data sets. Binary and fuzzy classifications may shed light on H/L classification ambiguity in MDR processes. Epistasis in populations of the Wellcome Trust Case Control Consortium was linked to coronary artery disease using two FCMEMDR assays.

Genomic researchers [44] claim that subtyping viruses is a serious challenge for the fields of virology and epidemiology. Subtyping viruses have received a lot of interest in the previous decade. Many viral subtyping methods narrow down to a certain virus family. There is a lot of confusion about how to define HIV and influenza, even among professionals. Convolutional Neural network-based (CNN) viral subtyping automation is possible with the viral genome deep classifier (VGDC). The approach may subtype any virus, as shown by testing on dengue, hepatitis B and C, HIV-1, and influenza A datasets. Each of the investigated virus types had an F1-score value between 0.85 and 1.00, with the exact range dependent on the virus type and the number of subtypes. In terms of HIV-1 and influenza A, VGDC is superior to CASTOR and COMET.

GWAS are used in case-control studies to find genetic variations associated with an observed association [45]. Phenotypic effects-carrying SNPs are being actively explored (*i.e*., disease traits). High-scoring SNPs in GWAS are typically those with low p-value sets. The approach is useful for discovering SNPs linked to illness susceptibility, despite the presence of some false positives. There is a lack of consensus on the optimal measures to prove genome-wide significance. Many people believe that studying SNP epistatic interactions and phenotypic expression will become feasible as p-values decline. Multifactor dimensionality reduction (MDR) uses this technique to isolate SNP combinations that have an effect on a given result. Higher-order combinations significantly increase processing complexity, making MDR more difficult. Understanding epistatic interactions in complex disorders is essential for precise genotype-phenotype mapping. Now, researchers detail a unique approach to GWAS case-control classification tasks through the extraction of higher-order SNP interactions from complex genotyped data. Stacking autoencoders, logistic regression, and quality control for GWAS are used to accomplish this. Researchers focus on defining subtypes of preterm birth, which have an unexplained heritability of 20 - 40% and are partly determined by genetics. Researchers use the dbGap GWAS dataset, which includes normal and preterm births to African-American women from low-income backgrounds. The epistatic interactions between SNP sequences were discovered using a stacked autoencoder model and then used to further develop a classifier for term and preterm deliveries. Each model is put through the standard battery of tests used to evaluate binary classifiers. Results show that a Fischer Linear Discriminant Analysis (FLDA) classifier model built from 4,666 raw SNPs obtained using logistic regression can achieve a 98.28% accuracy rate when applied to a variety of genomic data sets.

Genetic variations (GVs), as shown by research [46], may be useful for discerning illness-prone groups, determining populations at risk, and explaining disease propensity and response to therapy. Mainstream use of machine learning techniques for identifying GVs' nuanced phenotypic characteristics. It is possible that deep neural networks (DNNs) may learn non-linear mappings that transform GVs data into representations that are more amenable to grouping and classification than human feature selection. A learning algorithm's efficacy is influenced by a number of factors, including the quantity and quality of the data available and the accuracy of the representation used. To categorize people and estimate regional ethnicity from GVs, this research offers convolutional embedding networks (CEN) which combines two DNN architectures named Convolutional embedded clustering (CEC) and Convolutional autoencoder (CAE). Approximately 95 million GVs were utilized for CAE-based representation learning, with 2,504 individuals across 26 ethnicities represented in the “1000 genomes” dataset, and 279 people across 130 ethnicities represented in the “Simon’s genome diversity” dataset. In comparisons of quantitative and qualitative accuracy and scalability, their technique excels above VariantSpark and ADMIXTURE. In only 22 hours, CEC is able to cluster populations according to predetermined criteria with a clustering accuracy (ACC) of 89%, a normalized mutual information (NMI) of 0.92, and an adjusted rand index (ARI) of 0.91. The CAE classifier can correctly identify samples of unknown origin with an F1 score of 0.9004 and a Mathews correlation coefficient (MCC) score of 0.8245. Significant biomarkers are discovered using gradient-boosted trees (GBT) and Shapley Additive explanations (SHAP). Their approach is public, fast, and extensible from 5 to 100% of human genome sets.

BD-I and BD-II are different in terms of symptoms, diagnosis, and treatment, as reported by genomics Researchers [47]. Many people incorrectly believe that BD-II is only a subtype of BD-I. This study uses data science to identify SNPs that contribute to BD-I and BD-II categorizations. One hundred thirty-six Affymetrix Axiom Genome-Wide TWB Array Plates were used to conduct a screening and genotyping of Han Chinese. The AUC for the classifier created with 23 SNPs was 0.939, whereas the AUC for the classifier created with 42 SNPs was 0.9574, an increase of 1.8%. The percentage of correct classifications rose by 3.4%. The authors conduct a functional analysis of GO and Pathway, uncovering important factors like calcium ion binding, GABA-A receptor activity, the Rap1 signaling network, extracellular matrix proteoglycans, IL12-mediated signaling events, nicotine addiction, and the PI3K-Akt signaling pathway. SNP-SNP interactions may also be explored in addition to the standard SNP-finding process.

Genomic data sequences are capable of representing a wide variety of information about the underlying species. For instance, in human beings, the genomic sequences assist in the presence of cancer, diabetes, heart and stroke issues, and other diseases [48]. Genetic specialists claim that the SARS-CoV-2 virus, which was first detected in Wuhan, China, has already infected millions of people throughout the world [49]. It is important to determine whether a well-known virus or a novel virus is to blame whenever a new viral pandemic emerges. Researchers provide a deep learning method that employs CNN in conjunction with a bidirectional long short-term memory network (Bi-LSTM) as shown in Fig. (**5**) to categorize SARS CoV-2 among the Coronaviruses. In this article, they have categorized the regulatory characteristics of genomic sequences. Transcription factors are the mediators of gene expression. After extensive testing, it was determined that the proposed CNN-Bi-LSTM model successfully distinguished SARS CoV-2 from other Coronaviruses with an accuracy of 99.95%, an area under the receiver operating characteristic curve (AUC) of 100.00%, a specificity of 99.97%, a sensitivity of 99.97%, a Cohen's Kappa of 0.9978, and MCC of 0.9978. At its peak performance, CNN-Bi-LSTM can accurately identify putative regulatory motifs or binding sites 99.76% of the time with an ROC AUC of 100.00%, a specificity and sensitivity of 99.76%, a mean correlation coefficient (MCC) of 0.9980, and a Cohen's Kappa of 0.9970. These results support the use of deep learning strategies for the detection of SARS-CoV-2 and its regulatory motifs.

Copy number variations in human diseases like cancer may now be detected using cutting-edge methods like array comparative genomic hybridization (aCGH) [50]. DNA CNVs are useful for prognosis and monitoring because of the correlation between the illness they are associated with and the copy number variation. Machine learning models for classifying tissue types. The categorizing process is made more difficult by the fact that many biological characteristics have little to no bearing on diseases. Numerous feature selection algorithms have been developed for use in categorization domains. Researchers present a new feature selection approach based on structured sparsity-inducing norms to help find aCGH biomarkers that may be utilized to establish illness subtypes. The suggested method was tested using data from four openly available aCGH datasets. On a consistent basis, sparse learning-based feature selection achieves better results than other approaches. Researchers conduct an in-depth analysis of the aCGH biomarkers chosen using their method, and the data researchers find backs up their claims. Geneticists [51] believe that the 2019 SARS-CoV-2 was the spark that set off the COVID-19 epidemic.

Different methods of genomic analysis are required to fully understand this one-of-a-kind pathogen and how it interacts with others. In this study, the genetic fingerprints of SARS-CoV-2 and seven other viruses were studied by looking at their intrinsic dinucleotide sequences. Genome sequences were converted to dinucleotide relative frequencies using XGBoost, which allowed for their classification. Different SARS-CoV-2 sequences and data on all eight species were used to teach the classifiers how to distinguish between them. The eight-species classification was a complete success thanks to their method. SARS-CoV-2 and MERS-CoV have similar patterns of dinucleotides in their genomes, therefore the models achieved 86% balanced accuracy in classifying SARS-CoV-2 sequences into six continental zones, and 67% balanced accuracy in determining whether SARS-CoV-2 samples were from Asia. If you compare Oceania to other continents, you will see that it has a disproportionate number of TT dinucleotides and an abnormally low number of CG dinucleotides. Despite the large variation in other dinucleotide signatures, the vast majority of genomes showed similarities in the dinucleotide signatures of AC, AG, CA, CT, GA, GT, TC, and TG. Using relative dinucleotide frequencies, this study demonstrates how to differentiate between closely related species.

In order to get a deeper understanding of the dynamic rules that control proteins, cell biology, and disease processes, research into subcellular localization (SCL) of proteins and proteome variation in many human tissues and organs [52] is essential. There has been a lot of development in these two fields, but the question of how proteins are distributed throughout the body's organs remains unanswered. Physical PPIs and tissue-specific functional linkages were utilized to predict protein SCL on tissue specificity. Among the nine types of tissue-specific protein-protein interaction networks, researchers focused on eight using Bayesian collective Markov random fields (BCMRFs). The results prove the efficacy of their approach to identifying SCL in different tissues. Over a thousand and fourteen SCL-reliant proteins were found. Some of the 549 projected tissue-specific candidate proteins were verified using text mining.

This phenomenon, known as epistasis, sheds insight into the potential for connected networks of genetic variations to drive phenotypic manifestation, thereby expanding on the “common illness, common variation” notion. Pairwise and infinite-arity epistasis analyses are common when using variant networks, such as variant against variant or high-order interactions. Standard approaches, such as GWAS, increase the number of pre-tests when a false discovery rate (FDR) is already an issue. The FDR increases as the number of tests increases by a factorial rate. Epistasis may increase the computational load by a factor of O(n!); however, this mostly depends on the nature of the inquiry. This research [53] recommends a novel strategy for finding epistasis, one that makes use of linear classification (LC) methods and filtering best practices to emphasize interactions. The regularization of the significance and dependability of SNPs is accomplished by the use of random sampling, which divides and forms sample sets at random. Initial findings show that interaction detection may be performed rapidly. Through the finding of epistasis, researchers were able to identify eight risk candidate interactions between five variations and one protective variable in the classification of breast cancer patients.

According to a study [54], one of the trickiest parts of evaluating high-throughput genomic data is creating efficient computer techniques to find statistically significant SNPs. Single-locus analysis is used by GWAS to explore the association between SNPs and phenotypes. Unfortunately, genetic diversity in complex disorders is not considered by this strategy. A new set of methods is required for modelling SNP relationships. In order to discover epistatic SNP interactions, their method expands on GWAS by integrating stack autoencoders (SAEs) with association rule mining (ARM). For epistasis analysis, the most important SNPs are selected after quality control and association analysis of GWAS data. By modifying the level of backing and confidence it has in each rule, SAERMA affects the final multi-layer perceptron neural network (MLPNN's) ability to classify data. Most successfully, researchers were able to decrease the 204 SNPs to 100 units with 90% accuracy using 50 hidden units.

Epistatic interactions, or the nonlinear interacting effects of SNPs are argued to be an essential step in identifying the genetic roots of complex disorders by genome researchers [55]. Even though several techniques have been devised, the methodological and computational challenges are well-known. The detection strength of algorithms based on Ant Colony Optimization (ACOs) is higher, their temporal complexity is managed, and they use a heuristic positive feedback search. There is not yet a complete overview available. Twenty-five different ACO-based epistasis methods are investigated here. Epistatic interactions, as well as the ACO method for detecting them, are first presented. Next, Researchers take a look at four different perspectives on ACO-based methods for detecting epistatic interactions: route selection techniques, pheromone update suggestions, fitness functions, and two-stage designs. This research examines potential future directions in epistasis detection and evaluates the strengths and weaknesses of the technologies already available.

Recent advances in genome sequencing [56] have made it feasible to investigate the correlation between genetic variation in humans and illness for the first time. The cost of comprehensively genotyping a large cohort is substantial. Particularly in GWA research, imputation methods see extensive application. Genome Imputation makes use of advanced statistical methods. It is becoming more common to outsource imputation because of its data and computing requirements, however, this raises privacy concerns. Researchers take a look at how Machine learning (ML) and the Homomorphic encryption scheme (HES) may be used for fast, scalable, and private genotype imputation. For single-output multi-class classification, when nonlinear functions are computationally costly, ML-based privacy-preserving inference is the best option. Multiple types of outputs are produced from each genome, requiring optimization and/or approximation. By using HES, researchers modify linear models for genotyping imputation into confidential variants. Their privacy-preserving genotype imputation method obtains a micro area under curve score of up to 99.9% on real-world, large-scale datasets with as many as 80,000 targets.

Scientists who study genomes [57] say that Genome Wide Association (GWA) case-control data of complex illnesses with high dimensions renders many SNPs inappropriate. In Random Forest, a basic random sampling method will choose a large number of subspaces that have no statistically significant single nucleotide polymorphisms. There are times when a thorough search for the optimum m-try is required to ensure that relevant and informative SNPs are included while irrelevant ones are left out. GWA is very sluggish for multidimensional data. In this work, Researchers propose using a stratified sampling technique inside a random forest to pick out feature subspaces for high-dimensional GWA data. Developing a workable equal-width discretization strategy for SNPs is their primary focus. A subspace for a decision tree is created by randomly selecting a number of SNPs, with that number being the same for both groups. Using stratified sampling, Researchers can guarantee that each subspace has a significant number of informative SNPs without the astronomical processing costs associated with keeping a random forest and carefully looking for the best model. Researchers demonstrate the effectiveness of the proposed stratified sampling strategy by applying it to two sets of genome-wide SNP data, resulting in random forests with improved accuracy and a lower error limit than those generated using Breiman's approach (Parkinson case-control data with 408 803 SNPs and Alzheimer case-control data with 380 157 SNPs). With regard to Parkinson's information, researchers provide numerous fascinating genes that may be linked to neurological disorders.

Geneticists attest to Dengue's importance as an arthropod-borne disease [58]. The detection of dengue phenotypes by laboratory and patient testing is inconsistent. Researchers provide a machine-learning method for estimating the severity of dengue sickness using data from human genetics. Their study of 102 Brazilian dengue patients and controls included genotyping 322 SNPs associated with innate immunity. An ANN and SVM are used in their strategy to determine which subset of loci is most useful for classifying patients with dengue fever or severe dengue. The ANN was able to achieve 86% accuracy, 98% sensitivity, and 51% specificity after being trained on 13 important immunological SNPs using dominant or recessive models. The proposed categorization method based only on genetic markers may be able to identify those at high risk of obtaining a severe dengue phenotype even in uninfected locations. Their findings highlight the significance of genetic background in the emergence of the dengue phenotype. In fact, the suggested method may be used to treat a wide range of Mendelian and genetic disorders.

Human DNA encodes growth, physiological equilibrium, and the inheritance of features [59]. Every individual is unique because of our genetic variety. Individual differences may be investigated in terms of disease biology and pharmaceutical efforts to restore balance using genetic technology, among other possible therapeutic uses. A rare pathogenic mutation in a genome might provide a molecular diagnosis for patient management and family healthcare. Despite the increasing clinical use of unbiased genomic tests such as whole genome sequencing (WGS), clinical exome sequencing (cES), chromosome microarray analysis (CMA) with array comparative genomic hybridization (aCGH), or SNP arrays, molecular diagnoses will continue to necessitate clinical expertise and knowledge of each testing type. Increasing case-solving rates, functionally annotating a large portion of the human genome, and comprehending the genetic contributions to sickness will all benefit clinical genomics and precision medicine.

NGS has influenced how uncommon diseases are detected and treated, according to a study [60]. More genetic illnesses may now be detected and identified thanks to the development of new diagnostic technologies. Despite the availability of whole exome and whole genome sequencing, diagnostic success rates remain low. In 30% of cases, a molecular diagnosis is made. Because of the convergence of research and clinical diagnostic testing in national sequencing initiatives developed in the past five years, the range of genetic illnesses is growing. Clinical research for genetic sickness diagnosis is therefore often integrated with efforts to uncover candidate variants for new disease genes. At the molecular level, scientists examine the key obstacles confronting gene-disease diagnosis. Researchers examine the possible advantages of a gene-to-patient technique for speeding up the discovery of new genes, taking into consideration the impact of incomplete penetrance, non-coding variation, and structural alterations. The impact of incomplete penetrance, non-coding variation, and structural changes on the frequency of accurate diagnoses are being studied by researchers.

According to researchers [61], studies in genomics have revealed that the explosion of data is related to the huge decline in the price of sequencing. It is actually challenging to identify the particular mutations that generate phenotypes and diseases. Because of the National Institutes of Health (NIH's) Undiagnosed Diseases Network (UDN), progress has been made. 6,000-13,000 illnesses still have undetermined genes. Genetic research not only aids the over 400 million people worldwide who suffer from rare diseases, but it also advances our understanding of more prevalent disorders. Model organism platforms are valuable for revealing the pathogenicity of variation, uncovering new gene-disease connections, and obtaining an understanding of the underlying pathophysiological mechanisms that might lead to the development of novel therapies. To aid in the diagnosis, the UDN Model Organism Screening Center (MOSC) employs information technology and functional research in worms (Caenorhabditis elegans), flies (Drosophila melanogaster), and zebrafish (Danio rerio). The MOSC has shown benefits in identifying difficult situations, such as those with multiple organ dysfunction. Furthermore, the MOSC encourages diagnostic collaboration between fundamental scientists and medical practitioners. When creating tailored experimental approaches, aspects such as model organism appropriateness, gene and variation analysis, and patient presentation must all be addressed. MOSC also creates bioinformatics-related experimental reagents and tools. Many individuals from many backgrounds collaborated to make the MOSC a success. These individuals are knowledgeable in human and model organism genetics, and variant bioinformatics, and maintain continuing relationships with clinical teams. The NIH, rare illness family groups, charitable organizations, business partnerships, and other funding sources urge MOSCs to preserve and build their MOSC-like research institutes (MON).

Despite the fact that GWAS have revealed the genomic architecture of complex human features and illnesses [62], understanding the pathways that link genetic variation to pathophysiology remains challenging for many applications. To bridge this gap and put ideas to the test in practice, we must build techniques for doing so. Researchers used cross-phenotype links to collect common SNPs and calculate enrichment in order to discover traits with a similar genetic architecture. The same genetic structure has been studied clinically, cellularly, and molecularly. Researchers created a dynamic online database (Interactive Cross-phenotype Analysis of GWAS database (iCPAGdb)) that allows users to quickly examine and analyze GWAS summary information that they input. The database produced innovative insights for understanding sickness causation while also highlighting well-known phenotypic connections. The GWAS signals from severe COVID-19 overlapped significantly with clinical illnesses such as idiopathic pulmonary fibrosis caused by the DPP9 gene. DPP9 levels were higher in the transcriptomes of COVID-19 patients infected with SARS-CoV-2 compared to healthy controls and bacterial infections. Cross-phenotype SNPs connected to severe COVID-19 and other characteristics revealed that the GWAS signal at the ABO gene interacted with plasma protein levels of the SARS-CoV-2 receptor CD209 in people. This suggests that the degree to which COVID-19 sickness shows itself may be affected by ABO glycosylation of CD209.

The 100,000 Genomes Project in the United Kingdom delivered the first clinical genome sequencing service (100KGP). For statistical analysis, only gene panels customized to each individual patient were used [63]. Panels often rely on specific traits and may exclude alternative diagnoses that do not meet those criteria. The Researchers' technique enables the rapid identification of hazardous aberrations such as 100KGP misses. Low LOEUF (Loss-of-function Observed/Expected Upper-bound Fraction) readings indicate haploinsufficiency. DeNovoLOEUF searches for rare, de novo loss-of-function mutations with a LOEUF score of 0 in the sequencing data of 13,949 unusual disease trios in the 100KGP. According to patient diagnostic reports, DeNovoLOEUF discovered changes that were either diagnostic or partially diagnostic in 98% of instances (whereby the variant was responsible for some of the phenotype). In other words, 100KGP's routine analysis “omitted” 39 diagnoses that are now being offered to patients.

The ability of medical research to grasp the nature, course, and interaction of illness has been enhanced by genomic technologies [64]. Because of advances in quicker, cheaper sequencing technology, pathogen diversity can now be quantified with unparalleled precision and resolution. The 2019 coronavirus pandemic and modeling advancements that enable swift assessments of nascent epidemics to guide monitoring, coordination, and resource deployment demonstrate the growing use of models that can predict the development and extent of infectious disease outbreaks. Genetic research is mostly done in hindsight. These models are useful for studying disease variety and evolution, but they are not necessarily favorable to the development of successful therapeutics. Because they are at the heart of the most serious infectious public health challenges, interventions must address both virulence mechanisms and pathogen diversity. This perspective examines the intersection of these fields, discusses the challenges that surveillance specialists and modelers face, and provides recommendations for how to proceed with combining longitudinal genetic data with statistical learning and interpretable modeling to make accurate forecasts.

Rare diseases afflict 30 million Americans and 300-400 million people worldwide and may result in long-term illness and damage. To get to heuristic diagnoses, traditional diagnostic approaches that integrate in-person experience with published research are applied. Patients with difficult-to-diagnose diseases often die. Exome sequencing, microarrays, and gene panels have been used to find previously unknown disorders [65]. Because of these technologies, 25% to 35% of previously unidentified patients have received conclusive diagnoses. Many of these patients are undiagnosed. Scientists highlight numerous alternatives to standard exome sequencing in this review. Researchers underline the benefits of whole-genome sequencing, long-read technologies, methyl profiling, transcriptomics, metabolomics, proteomics, and pan-genome reference. Researchers stress computer-based technologies in order to identify people who have a genetic defect or a collection of desirable characteristics. This article also presented some advice to assist medical practitioners and researchers dealing with non-diagnostic exomes.

Next-generation sequencing has helped our understanding of mosaicism in genetic diseases, according to a genomics study reported [66]. PHACTR1, SCN8A, KCNT1, CDKL5, NEXMIF, CUX1, TSC2, GABRB2, and SMARCB1 all have variants. The authors discuss 11 cases in which mosaic changes revealed by genome sequencing (GS) and/or targeted gene panels (TGPs) were responsible for the proband's phenotype, and two cases in which mosaicism seemed to be inherited from both parents. There were three large duplications and three major deletions (UBE3A, GABRB3, and MAGEL2) (ARFGAP1, EEF1A2, CHRNA4, and KCNQ2). Everyone who participates in the NYCKidSeq project (a research program studying the communication of genomic information in clinical care) will help researchers learn more about the therapeutic usefulness and diagnostic yield of GS for children with suspected genetic diseases in New York City. Despite finding a link between the number of variant alleles and the intensity of symptoms, researchers were unable to detect mosaicism in tissues with significant clinical significance. Despite this, researchers saw an increase in GS identification. We were unable to compare the efficiency of GS and TGP in identifying mosaicism due to the limitations of our experiment. This case series provides recommendations for laboratory and clinical interpretation, providing support to the hypothesis that mosaicism plays a role in pediatric genetic disorders.

Outside of India, the Beak and feather disease virus (BFDV) has been recorded in Oceania, Africa, Asia, and Europe [67]. BFDV was discovered in the Trichoglossus haematodus rainbow lorikeet by Indian researchers. Phylogenetic analysis indicated that the Indian BFDV genome, Rep, and Cap sequences were most similar to those of T. haematodus, the pathogen that infected the BFDV in Australia. While the rainbow lorikeets were sick, the Indian and exotic Psittaciformes kept with the BFDV-infected lorikeets remained healthy and tested negative for the virus after four months. This discovery indicated that BFDV may transmit host-specific diseases. Researchers used phylogenetic analysis to divide the 361 BFDV genome sequences from various bird species into separate groups. The BFDV entire genome sequences found in T. haematodus have a lot of diversity, for example, in the Rep origin, the intergenic region between the 3′ ends of the Rep and Cap genes. According to the BFDV-host coevolution research, coevolution of the BFDV entire genome, Rep gene, Rep protein, Cap gene, and Cap protein sequences with their host avian species has resulted in the TimeTree of diverse Psittaciformes bird species. To the best of our knowledge, no research has shown that the BFDVs found in Trichoglossus sp. may infect other bird species. The BFDVs discovered in a foreign bird in India are unlikely to propagate to local Psittaciformes. Continuous BFDV monitoring in Indian birds may aid in the identification of the virus's DNA and the development of preventative strategies.

Machine learning and data mining are two approaches that have gained popularity in recent years for application in medical diagnostics, particularly in the context of human genome research [68]. SNPs have been related to a broad range of human disorders. There are many possible ways to distinguish sick SNP samples from healthy SNP samples. This study used Conditional mutual information maximization (CMIM) to identify the optimal SNPs for identifying hypertension illness. Linear Discriminant Analysis (LDA), Naive Bayes (NB), K-Nearest Neighbors (KNN), (ANN), and (SVM) were all investigated. The proposed approach was experimentally evaluated using supervised classification experiments, and the results showed that the ensemble approach using the SVM, 5-NN, and NB classifiers achieved the highest classification accuracy (93.21%) and F1 score (91.72%) overall, demonstrating that the approach was well-suited for detecting hypertension disease from SNPs data sets.

AD, the most common type of dementia, has no viable treatments or therapies at the moment [69]. Several researches have been conducted to investigate the various processes and causes of AD. Many researchers use gene expression data to investigate the genetics of illness and identify risk genes. Here, the researchers proposed a machine-learning strategy for discovering possible biomarker genes. Some of the 14 genes we discovered have been validated in scientific investigations. Several machine learning algorithms have been tested using the GSE5281 gene data.

Early illness detection is crucial since there are not enough hospitals [70]. Cancer survival rates are heavily influenced by how fast it is identified. Cancer is caused by thousands of distinct DNA mutations. Cancer tumor mutations are complicated and need much investigation. A molecular pathologist examines genetic variations manually. There are nine main types of clinical indication strips that we are aware of. This approach includes a multiclass classifier for categorizing genetic changes based on clinical symptoms. Texts are analyzed using Natural language processing (NLP) to look for signs of gene mutation. To characterize gene mutations, many machine learning approaches, including NB, LR, Linear SVM, and RF Classifier, were used to a dataset comprising of genetic alterations and clinical evidence given by pathologists or specialists. Methods are evaluated to determine which provides the best results. Machine learning models may be built using gene, variance, and text features. Variations in gene mutations may help to predict cancer risk and provide therapy recommendations.

AD has been connected to genome analysis [71]. The symptoms of AD are not malignant initially, but over time they become more severe. It is actually progressive, which means that the disease will deteriorate with time. According to GWAS, apolipoprotein E (APOE) has been related to an increased risk of acquiring AD. SNPs account for the overwhelming majority of DNA differences. SNPs are one of the disease's biomarkers. SNPs assist clinicians in learning about and diagnosing illnesses at an early stage. The goal of this project is to identify SNP biomarkers for AD that will allow for early diagnosis and prognosis. In this case, researchers employ machine learning methods to look for AD biomarkers. The learning methods NB, RF, LR, and SVM were used to evaluate all AD genetic data of neuroimaging initiative phase 1 (ADNI-1)/Whole-genome sequencing (WGS) datasets. In ADNI-1, the learning techniques NB, RF, SVM, and LR attained accuracies of 98.1%, 97.97%, 95.88%, and 83.0%, respectively. The findings suggest that categorization approaches might be utilized to aid in the early detection of AD.

According to a recent study [72], researchers face tough circumstances because of SARS-CoV-2's fast proliferation. A unique learning system that can learn a vast pattern with fewer input (coronavirus genome sequences) is needed immediately. When there is not enough sequencing data at the start of a pandemic, learning from limited training samples is crucial. To stop the virus's transmission, patients must be appropriately identified and quarantined. Machine learning and deep learning algorithms require a lot of training data to recognize the target infection among similar ones. Neurochaos Learning (NL) is a technique for categorizing coronavirus genomic sequences. Chaos and nonlinearity in biological brain networks inspired the NL paradigm. Leave one out cross-validation gives NL a 0.998 sensitivity, 0.999 specificity, and 0.998 accuracy for multiclass classification (SARS-CoV-2, Coronaviridae, Metapneumovirus, Rhinovirus, and Influenza). We can identify SARS-CoV-2 from SARS-CoV-1 with a 0.99 or higher macro F1-score. Using one training sample per class, 1,000 randomly generated training sessions were created. NL's effectiveness is compared to LR, RF, SVM, and NB classifiers. Combining chaotic feature engineering with other machine learning approaches should lead to innovative uses of NL in genome categorization scenarios.

A patient's treatment plan will soon be based on their unique genetic composition, ushering in the era of personalized medicine [73]. Understanding the genes that put individuals at risk, the uncommon genetic variants that play a role, and so on is critical for treating and avoiding common diseases. Our research aims to educate scientists, clinicians, and pharmacists about the genetic variants that contribute to sickness risk. Researchers describe a novel gene-SNP-disease-drug mobile database that may be accessed through a smartphone app. We seek to aggregate data from worldwide clinical and genomics databases such as Ensemble, GenCode, ClinVar, GeneCards, DISEASES, HGMD, OMIM, GTR, CNVD, and Novos into a single, mobile-friendly resource. Over 59,000 protein-coding and non-coding genes are represented in the database, as are over 67,000 germline SNPs, over a million somatic mutations reported for over 19,000 protein-coding genes spanning over 1,000 regions, over 80,000 International Classification of Disease (ICDs) codes, over 123,000 National Drug Code (NDCs), and over 100,000 gene-SNP-disease correlations. The Researchers propose a strategy for integrating genomic and phenotypic data for gene-based designer medicines, precise targeting of tumor molecular markers, appropriate drug therapy, disease susceptibility prediction, detection of uncommon conditions, and therapies.

A majority voting ensemble method has been explained [74] where they proposed machine learning methods to predict the possible presence of heart disease in humans. Here the models were trained using the real-life data of healthy and ill patients. The model classifies the patient based on the majority vote of several machine learning models. The learning methods and their respective accuracies are Stochastic Gradient Descent (SGD) Classifier 88%, KNN Classifier 87%, RF Classifier 87%, LR Classifier 87%, and Hard Voting Ensemble Method (HVEM) 90%. Hence HVEM provides high accuracy of heart disease detection.

Another approach is a deep learning method to identify the genetic variants in the classification of AD [75], this approach uses nonlinear transformations to extract features from high-dimensional data. Here a novel three-step approach called the Sliding Window Association Test (SWAT-CNN) is used for the identification of genetic variants that identify phenotype-related SNPs that can be applied to develop accurate disease classification models. SWAT-CNN, a novel deep learning–based genome-wide approach achieved an accuracy of 75% to identify AD-associated SNPs and a classification model for AD.

Deep learning methods have also been used to identify the T2D using the nucleotide signals [76], here the five-stage approach is used where the T2D-associated DNA sequences are digitized using the entropy-based technique. The further stages are to extract and select the features and classify them using the SVM and KNN methods. A combination model of the proposed Entropy-based technique, Residual Neural network (ResNet), and SVM achieved the highest accuracy rate of 99.09%.

The machine learning method combined with linear predictive coding (LPC) is used for the identification and classification of Covid-19 viruses [77, 78]. The LPC and signal processing techniques were used for data compression after which pattern recognition models were used for the detection and separation of covid samples from other virus case studies. The SVM classifier was used for the classification of the datasets that were obtained from different countries. This model was performed with an accuracy of 98%, this high accuracy can be used in the future for quantifying and digitizing medical big data information.

Thus, it can be observed that a wide variety of models are proposed for genome processing, and each of them varies in terms of their performance characteristics. Successful integration of these genomic processing models into clinical workflows and real-world applications requires a multidisciplinary approach, collaboration between researchers and healthcare providers, data accessibility and sharing adherence to ethical and legal standards, clinician training, and careful planning to address the challenges and barriers specific to each healthcare system or settings. A comparison of these models is discussed in the next section, which will assist readers in identifying optimal models for their performance-specific use cases.

## 3 EMPIRICAL ANALYSIS OF THE REVIEWED GENOME PROCESSING TECHNIQUES

From the elaborative discussion about existing genomic processing models, it can be observed that these techniques vary extensively in terms of their applicability, quantitative characteristics, and other performance measures. Thus, in this section, these models are compared in terms of their absolute Accuracy (A), Recall (R), Delay (D), Scalability (S), Computational Complexity (CC), and Suggested Application (SA) levels. This will assist readers in selecting optimal models for their performance-specific use cases. The absolute values of accuracy & recall are available in the respective research papers, and values for delay, scalability & computational complexity can be referred to from this section. These values are converted in the fuzzy ranges of Low Quantization (L=1), Medium Quantization (M=2), High Quantization (H=3), and Very High Quantization (VH=4) levels.

### 3.1 Accuracy for Various Diseases with their Suggested Applications

As per the strategy discussed above, the accuracy and suggested applications for various diseases can be observed in Table **1**.

Based on this evaluation in Table **1** and Fig. (**6**), it can be observed that the diseases have been classified according to their specifications. For Neurological disorders, it is observed that NB [71] is highly suited to identify AD. Similarly, for Parkinson's disease, PDG-NET [7] has more accuracy than others. For various cancer types, it is perceived that LMER [29] outperforms better than other models that work on the identification of different cancer types, Also Netboost (PCA SHC) [31] has achieved good accuracy for identifying Leukemia disease.

For identifying the COVID-19 virus, CNN-Bi-LSTM [49] has achieved the highest accuracy whereas VGDC CNN [44] also gives good accuracy for identifying multiple virus types. To study the relationship between multiple diseases, it is observed that GC [2] gives the best results which identifies the relationship between Diabetes and CAD.

It is observed from Table **1** that the Ensemble model works well for the classification of general diseases, an Ensemble model comprises of ResNet and SVM [76] gives the best results for diabetes, whereas an Ensemble model consisting of SVM,5-NN and NB [68] works best for Hypertension disorder. It is also noticed that CIMM SVM [41] has attained higher accuracy for the classification of complex diseases. There have been various research conducted on GWAS where ML-HES [56] gives the best results for predicting disease genes, NBS [40] gives the highest accuracy in identifying the relation between diseases and NIHO [19] and miRTMC [34] outperforms others in inspecting the Gene-network and RNA-Gene network respectively.

### 3.2 Recall, Delay, Scalability, and Computational Complexity of Genomic Models

Similarly, the performance of these models in terms of recall, delay, scalability, and computational complexity can be observed from Table **2** as follows,

From this evaluation, it can be observed that DST [47], GAN [28], VGDC CNN [44], CNN Bi-LSTM [49], XGBoost [51], DLLR [45], SIN [50], CEN [46], and NBS [40] showcase higher recall levels. Thus, they can be used for highly consistent classification use cases.

Similarly, it can be observed that DgSeq [5], Ensemble [12], PreEGS RF [17], and ML-HES [56] showcase lower delay levels, and thus can be used for high-speed genome processing use cases.

In terms of scalability levels, ModuleSim [6], NIHO [19], GAN [28], Netboost (PCA SHC) [31], AGDPM [39], VGDC CNN [44], CEN [46], CNN Bi-LSTM [49], and ML-HES [56] outperform others, thus can be used for large-scale dataset processing applications.

In terms of complexity, RAA [8] outperforms other models, and thus can be used for low-complexity processing use cases. Based on these observations, researchers can identify optimal models for their performance-specific use cases.

### 3.3 Genome Processing Efficiency Rank (GPER) of Genomic Models

Genome Processing Efficiency Rank (GPER) is a metric used to evaluate how efficiently a system or method processes genetic data, such as DNA sequences. It is calculated using a specific formula, which takes into account several important factors:

- **Accuracy (A):** Accuracy measures how close the results are to the true values. In the context of genetic data processing, this would indicate how well the system correctly identifies and interprets genetic information.
- **Recall (R):** Recall is a measure of how well the system identifies all relevant data points. In genomics, this could refer to how effectively the system captures all important genetic information without missing anything significant.
- **Computational Complexity (CC):** This factor reflects the computational resources required to process the genetic data samples. Higher computational complexity might indicate that the system needs more powerful computers or takes a longer time to complete its tasks.
- **Delay (D):** Delay represents any time delay in processing genetic data samples. A lower delay suggests that the system processes data quickly, which can be crucial in time-sensitive applications like medical diagnostics.
- **Scalability (S):** Scalability measures how well the system can handle increasing amounts of genetic data samples. Systems with good scalability can efficiently process both small and large data samples.

A novel Genome Processing Efficiency Rank (GPER) is evaluated *via* Eqn. (1),


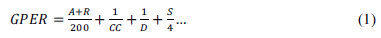


Based on this metric evaluation and Fig. (**7**), it can be observed that ML-HES [56], CEN [46], Netboost (PCA SHC) [31], ModuleSim [6], VGDC CNN [44], NIHO [19], AGDPM [39], PreEGS RF [17], CNN Bi-LSTM [49], DgSeq [5] outperform others in terms of accuracy, recall, scalability, computational complexity and delay levels, thus can be used for large-scale genome processing scenarios. Thus, researchers can select these models, and modify them as per their clinical use cases (Table **3**).

Researchers should also take into account some key ethical considerations and strategies for the genomic processing models such as Privacy and Data Security, Informed Consent, Data Bias and Fairness, Accountability and Transparency, and Clinical Validation. Addressing these ethical considerations and biases in genomic processing models requires a multi-faceted approach that involves technological, organizational, and regulatory measures. By prioritizing ethical principles, transparency, and fairness, the responsible integration of these models into clinical practice can be achieved while protecting patient rights and ensuring the highest standards of healthcare ethics.

## CONCLUSION AND FUTURE SCOPE

It is evident from the in-depth discussion of current genomic processing models that these methods differ greatly in terms of their applicability, quantitative features, and other performance metrics. Based on accuracy evaluation, it can be seen that PDG Net [7] achieves the highest accuracy for the identification of Parkinson's disease, NB [71] for AD types, and LMER [29] for different cancer types whereas Netboost (PCA SHC) [31] is best for identifying Leukemia disease. CNN-Bi-LSTM [49] is highly suited for identifying the COVID-19 virus, while GC [2] gives the best results for identifying the relationship between multiple diseases. An Ensemble model of ResNet and SVM [76] attained the highest accuracy for the identification of diabetes, whereas an Ensemble model of SVM,5-NN, and NB [68] is highly suited for identifying Hypertension disorder. CIMM SVM [41] is best suited for the classification of complex diseases.

DST [47], GAN [28], VGDC CNN [44], CNN Bi-LSTM [49], XGBoost [51], DLLR [45], SIN, GBT [46], and NBS [40] exhibit higher recall levels, according to recall evaluation. They can therefore be applied to use cases requiring highly consistent classification. Similar findings show that DgSeq [5], Ensemble [12], PreEGS RF [17], and ML-HES [56] exhibit lower delay levels and are suitable for use in high-speed genome processing use cases.

ModuleSim [6], NIHO [19], GAN [28], Netboost (PCA SHC) [31], AGDPM [39], VGDC CNN [44], CEN [46], CNN Bi-LSTM [49], MLPNN [54], and ML-HES [56] outperform others in terms of scalability levels and can be used for applications requiring the processing of large datasets. While RAA [8] outperforms other models in terms of complexity, making it suitable for use in low-complexity processing use cases. Researchers can choose the best models for their performance-specific use cases based on these observations. A novel Genome Processing Efficiency Rank (GPER) was evaluated to further streamline this model selection process. It was found that the following models outperform others in terms of accuracy, recall, scalability, complexity, and delay levels: ML-HES [56], GBT [46], Netboost (PCA SHC) [31], ModuleSim [6], VGDC CNN [44], NIHO [19], AGDPM [39], PreEGS RF [17], CNN Bi-LSTM [49], MLPNN [54].

The specific research gaps, challenges, limitations, and areas for improvement identified for genomic processing models are based on the analysis of Data Availability and Quality, Interpretable Models, Cross-Disease Predictions, Real-World Clinical Validation, as well as, Ethical and Privacy Concerns. Also, these can lead to the development of more robust and practical models for disease prediction and genomic analysis, ultimately benefiting both the research community and healthcare practitioners. In the future, researchers can also modify the identified models *via* the integration of bioinspired models like Genetic Algorithm (GA), Elephant Herding Optimization (EHO), Whale Optimization (WO), Grey Wolf Optimization (GWO), *etc*. This performance must be validated on larger datasets and can be improved *via* integration of transformer models like Auto Encoders, Q-Learning, *etc*. that will allow them to improve their accuracy incrementally w.r.t. real-time changes in datasets for clinical use cases.

## Figures and Tables

**Fig. (1):**
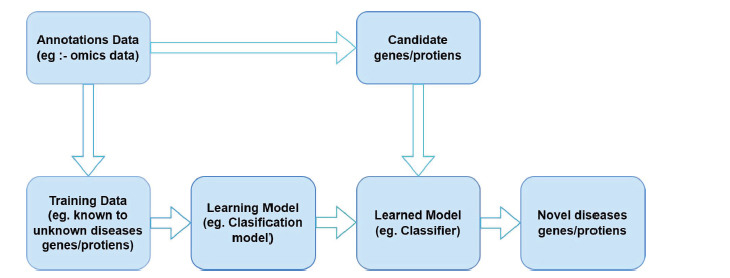
Design of a typical genome classification process [1-3].

**Fig. (2):**
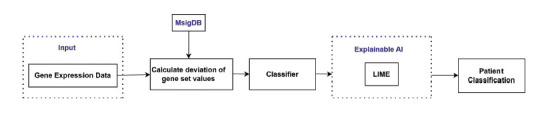
Analysis of alzheimer's patient using gene expression data [10].

**Fig. (3):**
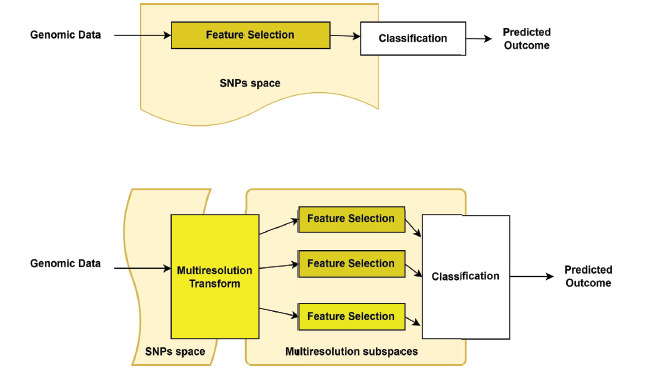
**Top Panel:** Typical pattern recognition workflow for predicting clinical outcomes from genomic data. **Bottom Panel:** Pattern recognition workflow proposed to work with genomic data, using a multiresolution analysis block as a pre-processing step [38].

**Fig. (4):**
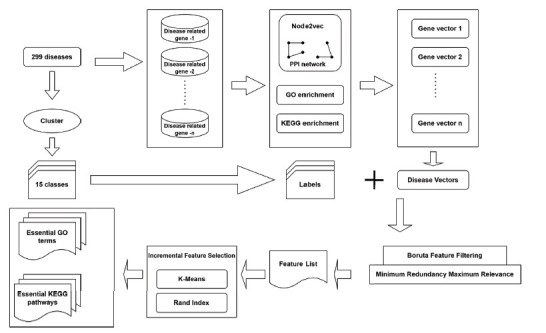
Derived gene vector from PPI network, GO, and KEGG enrichment. Feature selection method K-Means is incorporated to extract essential GO terms and KEGG pathways.

**Fig. (5):**
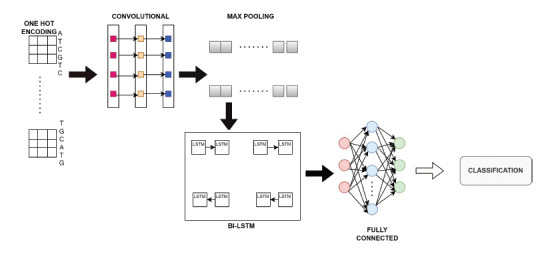
Schematic representation of the CNN-Bi-LSTM [49].

**Fig. (6):**
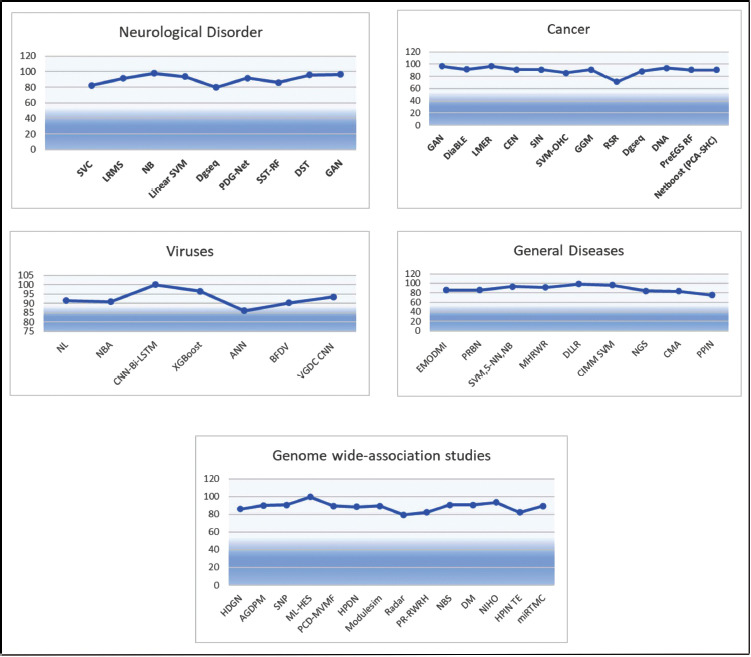
Accuracy of different genome processing techniques for various diseases.

**Fig. (7):**
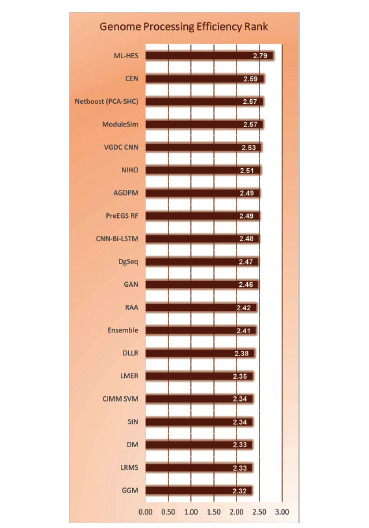
Top 20 genomic processing models that outperform in terms of accuracy, recall, scalability, computational complexity and delay levels.

**Table 1:** Accuracy comparison of genomic processing models along with their suggested disease type.

**Sr. No.**	**Disease Type**	**Disease Sub-Type**	**Model**	**Accuracy (%)**	**References**
1	Neurological disorders	Alzheimer's Disease (AD)	DgSeq	80	[5]
		-	SVC	82.4	[10]
		-	Ensemble	85.4	[12]
		-	LRMS	91.4	[38]
		-	Linear SVM	93.8	[69]
		-	NB	98.1	[71]
		-	SWAT-CNN	75	[75]
		Parkinson	PDG-Net	91.8	[7]
		-	SST-RF	85.9	[57]
		Huntington’s disease	GAN	96.4	[28]
		Bipolar	DST	95.7	[47]
2	Cancer	Ovarian	DNA	93.5	[1]
		Head, neck & kidney cell	DiaBLE	91.4	[20]
		Colorectal cancer	LMER	96.5	[29]
		Multiple cancer types	CEN	91	[46]
		-	SIN	90.9	[50]
		Breast	DgSeq	88	[5]
		-	GGM	90.8	[32]
		-	RSR	70.8	[61]
		Cancer tumor and its variants	SVM-OHC	85.5	[70]
		Leukemia	PreEGS RF	90.2	[17]
		-	Netboost (PCA-SHC)	90.5	[31]
3	Viruses	Covid-19	NBA	90.9	[35]
		-	CNN-Bi-LSTM	99.9	[49]
		-	XGBoost	96.5	[51]
		-	NL	91.5	[72]
		-	LPC-SVM	98	[78]
		Multiple virus types	VGDC CNN	93.5	[44]
		Dengue	ANN	86	[58]
		Beak and feather	BFDV	90.2	[67]
4	Relationship of multiple diseases	Diabetes, CAD	GC	91.4	[2]
		Brain, lung, asthma	RAA	83.5	[8]
		Thalassemia, diabetes, malaria, asthma	DELM	90	[14]
		Diabetes, bone, joint	dbGaP ensemble	86.5	[15]
5	Other diseases	Diabetes	PRBN	85.5	[9]
		-	ResNet, SVM	99.09	[76]
		Lung diseases	MHRWR	91.3	[11]
		Vitiligo	PPIN	75.4	[13]
		Asthma	EMODMI	86.02	[37]
		Complex disease	CIMM SVM	96	[41]
		Pre-term birth classification	DLLR	98.2	[45]
		Diagnosis of disease	CMA	83.5	[59]
		Rare disease	NGS	84.2	[60]
		Hypertension disorder	SVM, 5-NN, NB	93.2	[68]
		Heart disease	HEVM	90	[74]
6	Genome-wide association studies	Disease gene prediction	HDGN	86	[3]
		-	PCD-MVMF	89.4	[36]
		-	AGDPM	89.8	[39]
		-	ML-HES	99.5	[56]
		-	SNP	90.5	[73]
		Disease-disease relation	HPDN	88.5	[4]
		-	ModuleSim	89.4	[6]
		-	Radar	79.4	[16]
		-	PR-RWRH	82.2	[30]
		-	NBS	90.4	[40]
		Gene-Network analysis	DM	90.63	[18]
		-	NIHO	93.4	[19]
		-	HPIN TE	82	[42]
		RNA-gene network analysis	miRTMC	89.4	[34]

**Table 2:** Recall, delay, scalability, and computational complexity levels for different genome processing techniques.

**Sr. No.**	**Disease Sub Type**	**Method**	**Recall (%)**	**Delay**	**Scalability**	**Computational Complexity**	**References**
1	Ovarian	DNA	92.5	H	M	H	[1]
2	Diabetes, CAD	GC	88.7	H	M	H	[2]
3	Disease gene prediction	HDGN	87.25	H	H	H	[3]
4	Disease-disease relation	HPDN	88.25	H	H	VH	[4]
5	AD	DgSeq	88.70	M	H	H	[5]
6	Disease-disease relation	ModuleSim	90.60	H	VH	H	[6]
7	Parkinson	PDG-Net	87.7	VH	H	H	[7]
8	Brain, lung, asthma	RAA	84.5	H	H	M	[8]
9	Diabetes	PRBN	87.95	H	M	H	[9]
10	AD	SVC	90.85	VH	M	H	[10]
11	Lung diseases	MHRWR	88.4	H	H	H	[11]
12	AD	Ensemble	80.40	M	H	H	[12]
13	Thalassemia, diabetes, malaria, asthma	DELM	90.2	VH	H	VH	[14]
14	Diabetes, bone, joint	dbGaP Ensemble	83.0	VH	H	VH	[15]
15	Leukaemia	PreEGS RF	90.40	M	H	H	[17]
16	Gene-network analysis	DM	92.00	H	H	H	[18]
17	Gene-network analysis	NIHO	92.4	H	VH	VH	[19]
18	Head, neck and kidney cell	DiaBLE	89.0	VH	H	H	[20]
19	Huntington’s disease	GAN	96.5	VH	VH	VH	[28]
20	Colorectal cancer	LMER	89.4	H	H	H	[29]
21	Disease-disease relation	PR-RWRH	86.35	H	H	H	[30]
22	Leukemia	Netboost (PCA-SHC)	90.7	H	VH	H	[31]
23	Breast	GGM	90.1	H	H	H	[32]
24	RNA-gene network analysis	miRTMC	90.2	VH	H	VH	[34]
25	Disease gene prediction	PCD-MVMF	87.35	H	H	H	[36]
26	Asthma	EMODMI	88.4	H	H	VH	[37]
27	AD	LRMS	91.15	H	H	H	[38]
28	Disease gene prediction	AGDPM	90.65	H	VH	VH	[39]
29	Disease-disease relation	NBS	93.2	H	M	H	[40]
30	Complex disease	CIMM SVM	89.0	H	H	H	[41]
31	Gene-network analysis	HPIN TE	73.70	H	H	H	[42]
32	Multiple virus types	VGDC CNN	95.9	VH	VH	H	[44]
33	Pre-term birth classification`	DLLR	94.6	H	H	H	[45]
34	Multiple cancer types	CEN	93.4	H	VH	H	[46]
35	Bipolar	DST	97.7	H	M	H	[47]
36	Covid-19	CNN-Bi-LSTM	95.3	VH	VH	VH	[49]
37	Multiple cancer types	SIN	93.70	H	H	H	[50]
38	Covid-19	XGBoost	94.8	VH	H	H	[51]
39	Disease gene prediction	ML-HES	92.7	M	VH	H	[56]
40	Parkinson	SST-RF	86.0	H	H	H	[57]
41	Dengue	ANN	81.5	VH	H	H	[58]
42	Diagnosis of disease	CMA	75.4	H	M	H	[59]
43	Rare disease	NGS	70.5	H	L	H	[60]
44	Beak and feather	BFDV	85.5	VH	L	H	[67]
45	Hypertension disorder	SVM,5-NN, NB	90.2	H	M	H	[68]
46	AD	Linear SVM	90.2	H	M	H	[69]
47	Cancer tumor	SVM-OHC	80.5	H	L	VH	[70]
48	AD	NB	75.4	H	M	H	[71]
49	Disease gene prediction	SNP	85.4	H	H	H	[73]

**Table 3:** Genome Processing Efficiency Rank (GPER) for different genomic processing models.

**Sr. No.**	**Genomic Processing Model**	**GPER**	**References**
1	DNA	2.10	[1]
2	GC	2.07	[2]
3	HDGN	2.28	[3]
4	HPDN	2.22	[4]
5	DgSeq	2.47	[5]
6	ModuleSim	2.57	[6]
7	PDG-Net	2.23	[7]
8	RAA	2.42	[8]
9	PRBN	2.03	[9]
10	SVC	1.99	[10]
11	MHRWR	2.31	[11]
12	Ensemble	2.41	[12]
13	DELM	2.17	[14]
14	PreEGS RF	2.49	[17]
15	DM	2.33	[18]
16	NIHO	2.51	[19]
17	DiaBLE	2.24	[20]
18	GAN	2.46	[28]
19	LMER	2.35	[29]
20	PR-RWRH	2.26	[30]
21	Netboost (PCA-SHC)	2.57	[31]
22	GGM	2.32	[32]
23	LRMS	2.33	[38]
24	AGDPM	2.49	[39]
25	CIMM SVM	2.34	[41]
26	HPIN TE	2.20	[42]
27	VGDC CNN	2.53	[44]
28	DLLR	2.38	[45]
29	CEN	2.59	[46]
30	CNN-Bi-LSTM	2.48	[49]
31	SIN	2.34	[50]
32	XGBoost	2.29	[51]
33	ML-HES	2.79	[56]
34	SST-RF	2.28	[57]
35	ANN	2.17	[58]
36	CMA	1.96	[59]
37	NGS	1.69	[60]
38	BFDV	1.71	[67]
39	SVM,5-NN, NB	2.08	[68]
40	SNP	2.29	[73]

## LIST OF ABBREVIATIONS

ACC: Clustering Accuracy
aCGH: Array Comparative Genomic Hybridization
ACOs: Ant Colony Optimization
AD: Alzheimer's Disease
ADMM: Alternating Direction Method of Multipliers
ADNI-1: AD Genetic Data of Neuroimaging Initiative Phase 1
AGDPM: Advance Genome Disorder Prediction Model
AML: Acute Myeloid Leukaemia
ANN: Artificial Neural Network
APOE: Apolipoprotein E
ARI: Adjusted Rand Index
ARM: Association Rule Mining
AUC: Area Under the Curve
BCMRFs: Bayesian Collective Markov Random Fields
BD: Bipolar Disorder
BFDV: Beak and Feather Disease Virus
Bi-LSTM: Bidirectional Long Short-term Memory Network
CAD: Coronary Artery Disease
CAE: Convolutional Autoencoder
CEC: Convolutional Embedded Clustering
CEN: Convolutional Embedding Networks
cES: Clinical Exome Sequencing
CIMM: Conditional Mutual Information Maximization
circRNA: Circular RNA
CMA: Chromosome Microarray Analysis
CMIM: Conditional Mutual Information Maximization
CNN: Convolutional Neural Network
CNN: Convolutional Neural network
DAE: Denoising Auto-encoder
dbGaP: Database of Genotypes and Phenotypes
DDA: Drug Disease Association
DEG: Database of Essential Genes
DELM: Deep Extreme Learning Machine
DiaBLE: DIAMOnD Background Local Expansion
DMs: Disease Modules
DNA: Differential Network Analysis
DNNs: Deep neural Networks
EOT: Evolutionary Optimization Technique
FCME: fuzzy c-means-Based Entropy
FDR: False Discovery Rate
FLDA: Fischer Linear Discriminant Analysis
GAN: Generative Adversarial Network
GBT: Gradient Boosted Trees
GC: Graph Clustering
GGM: Gaussian-based Graphical Model
GNs: Gene Connections
GO: Gene Ontology
GPER: Genome Processing Efficiency Rank
GRN: Gene Regulatory Network
GS: Genome Sequencing
GVs: Genetic Variations
GWA: Genome Wide Association
GWAS: Genome-wide Association Studies
HDGN: Heterogeneous Disease Related Gene
HES: Homomorphic Encryption Scheme
HK: Housekeeping
HPDN: Human Pathway-based Disease Network
HPIN: Human Protein Interaction Network
HVEM: Hard Voting Ensemble Method
iCPAGdb: Interactive Cross-phenotype Analysis of GWAS Database
KEGG: Kyoto Encyclopedia of Genes and Genomes
KNN: k-Nearest Neighbour
LC: Linear Classification
LDA: Linear Discriminant Analysis
LIME: Locally Interpretable, Model-independent Explanations
LMER: Linear Mixed-effects Regression Model
lncRNAs: Long Noncoding RNAs
LOAD: Late-onset Alzheimer’s Disease
LOEUF: Loss-of-function Observed/Expected Upper-bound Fraction
LPC: Linear Predictive Coding
LR: Likelihood Ratio
LR: Logistic Regression
LRMS: Logistic Regression on Multiresolution Spaces
MCC: Mean Correlation Coefficient
MCC: Mathews Correlation Coefficient
MDR: Multifactor Dimensionality Reduction
MDR: Multifactor Dimensionality Reduction
MGP: Meta-path Gene Ontology Profiles
mi-RNA: MicroRNA
miRTMC: miRNA Target Prediction Using Matrix Completion
ML: Machine Learning
MLP: Multilayer Perceptron
MLPNN's: Multi-layer Perceptron Neural Network
MOSC: Model Organism Screening Center
MS: Metabolic Syndrome
MVMF: Metapath2vec++ and Matrix Factorization
NB: Naive Bayes
NBS: Network-based Sickness Module
ncRNAs: Non-coding RNA’s
NIH's: National Institutes of Health
NIHO: Neural Integration of Heterogeneous Data
NL: Neurochaos Learning
NLP: Natural Language Processing
NMI: Normalized Mutual Information
OMIM: Online Mendelian Inheritance in Man
PCA: Principal Components Aggregation
PCD: Predicting circRNA-disease Connections
PDGNet: Predicting Disease Genes Using a Deep Neural Network with Multi-View Features
PPIN: Protein-protein Interactions Network
PR: Page Rank
PreEGS: Prediction of Essential Genes in Comparison States
PreEGSRF: PreEGS variant based on the Random Forests Model
RAA: Resource Allocation-based Approach
ResNet: Residual Neural Network
RF: Random Forest
RNA: Ribonucleic Acid
ROC: Receiver Operating Characteristic
RWRDGN: Random Walk with Restart on a Reconstructed Heterogeneous Disease-gene Network
RWRH: Random Walk with Restart on Heterogeneous Network
SAEs: Stack Autoencoders
SCL: Subcellular Localization
SGD: Stochastic Gradient Descent
SHAP: SHapley Additive Explanations
SHC: Sparse Hierarchical Clustering
SNP: Single-nucleotide Polymorphism
SVC: Support Vector Classifier
SVM: Support Vector Machine
SWAT: Sliding Window Association Test
T2D: Type 2 Diabetes
TE: Enriched in Tissues
TF: Transcription Factors
TGPs: Targeted Gene Panels
UDN: Undiagnosed Diseases Network
VGDC: Viral Genome Deep Classifier
WGS: Whole Genome Sequencing
WGS: Whole-genome Sequencing
XAI: Explainable Artificial Intelligence
XGboost: Extreme Gradient Boosting
