# Supplementary material for: A Study and Analysis of Disease Identification using Genomic Sequence Processing Models: An Empirical Review
Source: Curr Genomics. 2023 Dec 12;24(4):207–35. doi: 10.2174/0113892029269523231101051455 (PMC10758128; doi:10.2174/0113892029269523231101051455)
Supplement: Supplementary file 1 — Supplementary material is available on the publisher’s website along with the published article. [file CG-24-207_SD1.pdf]

Supplementary Material

A Study and Analysis of Disease Identification using Genomic Sequence Processing Models: An Empirical Review

Sony K. Ahuja[1], Deepti D. Shrimankar[1,*] and Aditi R. Durge[1]

[1]*Visvesvaraya National Institute of Technology, Computer Science and Engineering, India*

DATASETS AND ITS CHARACTERISTICS

SR NO	DATASET	DISEASE TYPE	CHARACTERISTICS
1.	The Cancer Genome Atlas Program (TCGA)<br><br>The Cancer Genome Atlas (TCGA), a landmark cancer genomics program, molecularly characterized over 20,000 primary cancer and matched normal samples spanning 33 cancer types.	Ovarian Cancer	Gene expression and gene mutation data are collected from TCGA. The gene expression data sets (Agilent 244K Custom Gene Expression G450) and gene mutation data sets are downloaded using the TCGA2STAT package. (Version: March 2, 2016).
		Colorectal Cancer	Gene expression profiles of colorectal cancer patients is collected from the TCGA database and statistical analysis is performed based on the data. Three clinical variables (stage, gender and age) are compared with gene expression profiles, and it is found that stage was the most influential variables on gene expression.
		Leukaemia	Algorithm is applied to DNA methylation and gene expression data from The Cancer Genome Atlas (TCGA) Acute Myeloid Leukemia cohort to predict survival.
		Breast Cancer	Gene expression data sets of breast cancer is collected from The Cancer Genome Atlas which quantify the activity of genes from different cancer stages. There are four main progression stages, i to iv, of breast cancer included in the TCGA data sets. The gene expression levels of 17280 genes (Affymetrix HT Human Genome U133 Array Plate Set) for 496 breast cancers were used.
2.	Long noncoding RNAs (lncRNAs) database.<br><br>The LncRNADisease database is not only a resource that curated the experimentally supported lncRNA-disease association data but also a platform that integrated tool(s) for predicting novel lncRNA-disease associations.	Lung cancer	The latest version of lncRNA-disease associations was downloaded from the LncRNADisease database. After removing the duplicate data, a gold-standard dataset was obtained, which included 855 experimentally validated LncRNADisease associations. (including 472 lncRNAs and 130 diseases)

3.	A database of gene disease associations (DisGeNet)<br><br>DisGeNET integrates data from expert curated repositories, GWAS catalogues, animal models and the scientific literature. Additionally, several original metrics are provided to assist the prioritization of genotype–phenotype relationships. The current version of DisGeNET (v7.0) contains 1,134,942 gene-disease associations (GDAs), between 21,671 genes and 30,170 diseases, disorders, traits, and clinical or abnormal human phenotypes, and 369,554 variant-disease associations (VDAs), between 194,515 variants and 14,155 diseases, traits, and phenotypes.	Parkinson	130,820 disease-gene associations between 13,074 diseases and 8,947 genes were collected from DisGeNet database, which integrated disease-gene associations from multiple databases, e.g., PsyGeNET, ClinVar, OrphaNet, and the GWAS Catalog
4.	National Center for Biotechnology Information (NCBI)<br><br>The National Center for Biotechnology Information (NCBI) is part of the United States National Library of Medicine (NLM), a branch of the National Institutes of Health (NIH).<br>The NCBI houses a series of databases relevant to biotechnology and biomedicine and is an important resource for bioinformatics tools and services. Major databases include GenBank for DNA sequences and PubMed, a bibliographic database for biomedical literature.<br><br>NCBI Gene Expression Omnibus (GEO)<br><br>The Gene Expression Omnibus (GEO) database is an international public repository that archives and freely distributes high-throughput gene expression and other functional genomics data sets.	Diabetes<br><br><br><br><br>COVID 19	Raw data was collected from the Gene Expression Omnibus (GEO) of the National Center for Biotechnology Information (NCBI) (http://www.ncbi.nlm.nih.gov/geo/) and EBI array express (https://www.ebi.ac.uk/arrayexpress/). 5 large human gene expression datasets were used in this study; these datasets had accession numbers E-MTAB-5060, GSE114007, GSE55457, GSE7158, and E-GEOD-71595, where E-MTAB-5060 is an RNAseq dataset obtained from pancreatic islet cell studies of healthy and T2D individuals. GSE114007 is an RNAseq dataset derived from OA and normal human knee articular cartilage. GSE55457 is an Affymetrix DNA array dataset from a study of normal, OA and RA synovial tissues; GSE7158 is an Affymetrix DNA array-based study of blood cells from outlier high and low peak bone mass females aged 20-45; E-GEOD-71595 was from an RNAseq study of CD4+ cells obtained from auto-immune/inflammatory type JIA patients and healthy controls.<br><br>The dataset for classifying SARS CoV-2 genes amongst Coronaviruses was obtained from the NCBI genes database resulting in 329 unique sequences.

		Leukaemia	Both the training and test datasets include pairs of contrasted biomolecular networks along with the gene expression data for each gene. The expression data of each gene were collected from databases, such as the Gene Expression Omnibus (GEO) (https:// www.ncbi.nlm.nih.gov/geo/).
		Asthma	Two asthma gene expression datasets are used in the experiments, which are downloaded from the gene expression omnibus (GEO) repository. The first dataset (ID: GSE43696) contains 88 disease samples and 20 control samples, and each sample includes the expression data of 9194 genes. The second dataset (ID: GSE31773) contains 24 disease samples and 16 control samples, and each sample includes the expression data of 8789 genes.
		Multiple Disease types	Five SNP microarray datasets were used which are publicly available from NCBI GEO.<br>GSE67047 series includes SNP data from patients who have sporadic Medullary Thyroid Cancer (sMTC) and juvenile Papillary Thyroid Cancer (PTC). There are 225 individuals in the data set, 96 patients and 129 parents, and approximately 1,000,000 features. GSE9222 series includes SNP data associated with Autism. The dataset consists of 567 individuals, 335 patients and 232 parents, and more than 250,000 features. GSE34678 series includes SNP data from patients who have Colorectal Cancer. This study utilizes 124 samples, 62 as a case and 62 as a control and approximately 250,000 features. GSE13117 series includes SNP data associated with Mental Retardation taken from affected patients and their healthy parents. The dataset consists of 360 individuals, 120 children and 240 parents, and nearly 250,000 features. GSE16619 series includes SNP data related to Breast Cancer with more than 500.000 SNPs. This study used 111 individuals, 69 as cases and 42 as controls.

5.	NCBI Virus Database<br><br>NCBI Virus database is an integrative, value-added resource supporting retrieval, display and analysis of a curated collection of virus sequences and large sequence datasets.	Viruses	Virus genomic sequences were retrieved from publicly available databases. In particular, five datasets, each representing one virus type, were considered. These datasets represent the viruses of Dengue, Hepatitis B, Hepatitis C, HIV-1, and Influenza A. The Dengue virus sequences were downloaded from the National Center for Biotechnology Information (NCBI) virus database. The same database was the source of Influenza genomes. The genomic sequences of Hepatitis B were downloaded from The Hepatitis B Virus Database (HBVdb) while Hepatitis C genomic sequences came from the database of Los Alamos National Laboratory (LANL). The latter database was also the source of HIV-1 genomes.
6.	The database of Genotypes and Phenotypes (dbGap)<br><br>The database of Genotypes and Phenotypes (dbGaP) was developed to archive and distribute the data and results from studies that have investigated the interaction of genotype and phenotype in Humans.	Pre-term birth classification	The data, for this study, was obtained through authorised access to dbGap (Study Accession: phs000332.v3.p2). The dataset includes 722 cases and 1,057 controls. Cases were drawn from deliveries at the Boston Medical Center (BMC) that occurred before 37 weeks of gestation irrespective of birth weight. Controls include mothers who delivered term babies after 37 weeks of gestation also from the BMC cohort.
7.	National Taiwan University Hospital and the National Cheng Kung University Hospital.	Bipolar disorder BD-I and BD-II	The participants were 316 Han Chinese patients (195 with BD-I and 121 with BD-II) recruited from outpatient and inpatient settings
8.	The Hypertension (HT) dataset of the Wellcome Trust Case-Control Consortium (WTCCC)<br><br>The Wellcome Trust Case Control Consortium (abbreviated WTCCC) is a collaboration between fifty research groups in the United Kingdom in the field of human genetics. Established in 2005, the WTCCC aims to conduct genome-wide association studies (GWASs) to shed light on the genetic architecture of common human diseases.	Hypertension	2,001 samples from individuals with hypertension disease were retrieved from the Hypertension (HT) dataset of the Wellcome Trust Case-Control Consortium (WTCCC), while 1,500 samples of control (healthy) individuals were retrieved from the UK National Blood Service Control Group (NBS)

9.	GSE5281 brain dataset	Alzheimer's disease	The dataset contains 161 samples out of which 87 are diagnosed with Alzheimer's disease and 74 samples are from the healthy control group.
10.	Alzheimer's Disease Neuroimaging Initiative (ADNI) database.<br><br>ADNI is a multisite study that aims to improve clinical trials for the prevention and treatment of Alzheimer's disease.	Alzheimer's disease	Data was gathered from the ADNI database. ADNI Phase 1 data were collected from subjects (214 Non-AD and 177 AD cases). ADNI-1 samples were genotyped using the Human 610-Quad BeadChip, resulting in 620,901 SNPs.
11.	The University of California Irvine (UCI) repository.<br><br>The UCI Repository is a collection of real-life data, domain theories, and data generators that are used by the machine learning community for the empirical analysis of machine learning algorithms.	Heart Disease	The UCI repository was used to retrieve the heart disease database. The original database contains 76 attributes, but based on extensive experiments it was found that the most effective attributes were 14. The database contains the most dominant 14 attributes, which why they were chosen for training the model.
